# Accuracy of Freehand, Static, and Dynamic Computer‐Assisted Implant Placement: A Systematic Review and Meta‐Analysis

**DOI:** 10.1111/jre.70047

**Published:** 2025-11-27

**Authors:** Florian Sebastian Reiff, Annika Kroeger, Stefan Roehling, Christine Reiff, Moritz Kebschull

**Affiliations:** ^1^ Doctoral School of DTMD University Wiltz Luxembourg; ^2^ Straumann Group Freiburg im Breisgau Germany; ^3^ Department of Oral Surgery, School of Dentistry, Institute of Clinical Sciences, College of Medical & Dental Sciences The University of Birmingham Birmingham UK; ^4^ Birmingham Community Healthcare NHS Trust Birmingham UK; ^5^ Clinic for Oral and Cranio‐Maxillofacial Surgery University Hospital Basel Basel Switzerland; ^6^ Swiss MAM Research Group, Department of Biomedical Engineering University of Basel Allschwil Switzerland; ^7^ Private Dental Clinic “Oralchirurgie T1”, PD Dr. Gahlert & PD Dr. Roehling Munich Germany; ^8^ Technische Hochschule Mittelhessen Gießen Germany; ^9^ Division of Periodontology and Oral Rehabilitation, Dentistry, School of Health Sciences, College of Medicine and Health University of Birmingham Birmingham UK; ^10^ Periodontal Research Group, Dentistry, School of Health Sciences, College of Medicine and Health University of Birmingham Birmingham UK; ^11^ Birmingham NIHR Biomedical Research Centre University of Birmingham Birmingham UK; ^12^ Division of Periodontics, Section of Oral, Diagnostic and Rehabilitation Sciences, Columbia College of Dental Medicine Columbia University New York New York USA

**Keywords:** accuracy, dental implant, dynamic navigation, meta‐analysis, static navigation, systematic review

## Abstract

**Aims:**

Although static navigation (sCAIS) has been shown to be a well‐established technique in clinical practice, innovative methods such as dynamic navigation (dCAIS) are currently on the rise. The objective of this systematic review is to compare the accuracy of freehand (FH), sCAIS, and dCAIS.

**Methods:**

Following PRISMA guidelines, a systematic review and meta‐analysis with electronic and manual searches for comparative studies on transfer accuracy (TA) of freehand dental implant placement versus sCAIS versus dCAIS in pre‐clinical and clinical studies was conducted. The main outcome was TA between planned and placed implant positions, measured via axial, global coronal, and global apical deviations reported as mean values with standard deviation.

**Results:**

A total of 37 of 4511 screened articles were included, reporting data on 3104 implants across 606 models and 801 patients. Clinical studies revealed no statistically significant differences in TA between dCAIS and sCAIS. The pooled mean deviations were as follows: axial deviation −0.09° [95% CI: −0.66 to 0.48; PI: −2.11; 1.93], global coronal deviation −0.03 mm [95% CI: −0.16 to 0.11; PI: −0.47; 0.42], and global apical deviation −0.24 mm [95% CI: −0.48 to 0.00; PI: −1.12; 0.64]. However, subgroup analysis including RCTs only demonstrated that both sCAIS and dCAIS achieved statistically significantly higher accuracy in all investigated deviation parameters compared to the freehand protocol.

**Conclusion:**

Both static and dynamic computer‐assisted implant placement techniques are considered reliable methods for enhancing accurate implant positioning, offering significantly greater accuracy compared to the conventional freehand approach. The presence of bias risks and notable heterogeneity in the included investigations warrants careful consideration of the results.

## Graphical abstract

This meta‐analysis finds that both static and dynamic computer‐assisted implant placement methods offer significantly better transfer accuracy than freehand placement. No clinically relevant accuracy differences were found between static and dynamic approaches. Indicative safety margins for pre‐operative planning were derived based on pooled coronal, apical, and axial deviations.

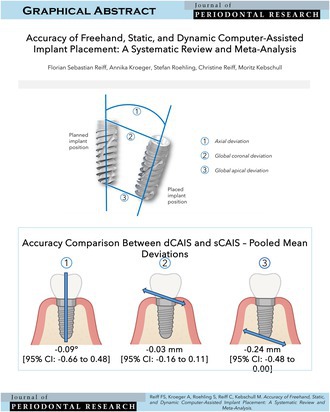

## 1 Introduction

The objective of dental implant therapy is to restore the functionality of the masticatory organ. It is defined as the anchoring of alloplastic materials in the jaw to create holding and supporting elements for the replacement of lost masticatory units [1]. One of the critical determinants of therapy success is the achievement of an optimal implant position, taking into account the patient's anatomy [2, 3]. In addition to other factors, this has an influence on the success rate and longevity of the implant and functionality of the prosthetic reconstruction [4, 5, 6, 7, 8, 9, 10, 11, 12].

Conventionally dental implant placement takes place after radiographic assessment in a freehand manner. In the context of digitalization, surgeons have access to computer‐assisted navigation systems that potentially facilitate optimal implant positioning [3]. With these systems, clinicians can use software to conduct pre‐operative virtual planning of the implant position, which can then be implemented in the surgical site using navigation technology [13]. The utilization of this technology allows the surgeon to be guided by a navigation system throughout the implantation process, which is why it is referred to as ‘guided surgery’ in this context. A variety of navigation techniques are available for clinical implementation [14]: (i) Static computer‐aided implant surgery (sCAIS) and (ii) dynamic computer‐aided implant surgery (dCAIS). sCAIS has been in use clinically for several years and has been the subject of extensive scientific investigation [15, 16, 17]. It is mentioned as the gold standard for guided surgery [18]. The more innovative method, dynamic computer‐aided implant surgery (dCAIS), is comparatively rarely used and scientific investigations relating to this method are sparse [19]. When using sCAIS, surgical instruments are mechanically guided by a prefabricated drilling template. In contrast, dCAIS enables real‐time monitoring of the instrument position during implant surgery using optical systems and allows immediate adjustments to be made in the event of deviations from the previously planned implant position. In the freehand method, the surgeon places the implant without any guiding system, relying solely on clinical experience and anatomical landmarks.

**FIGURE 1:**
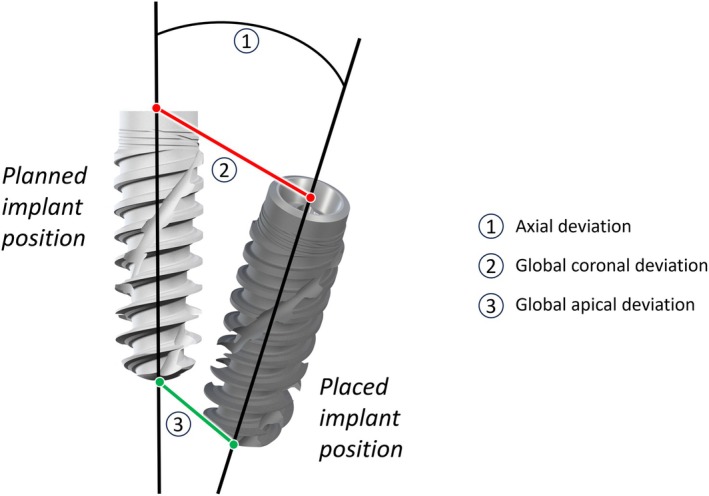
Illustration of the parameters indicating implant placement deviations. Assessed parameters of implant deviations are (1) axial deviation, (2) global coronal deviation and (3) global apical deviation.

The term ‘accuracy’ is a decisive set of deviation parameters identified as a critical indicator of the reliability of sCAIS and dCAIS [15]. This assesses the degree of accuracy with which the virtually planned implant position is transferred to the clinical surgical site. In this review, the discrepancy between the planned and the placed implant position is defined as transfer accuracy (TA). The TA is assessed via measurement values of the axial deviation (in degrees) of the longitudinal axis of the implant, as well as the maximum global deviation at the coronal implant base and at the apical tip (in millimeters) of the implant, as demonstrated in Figure 1. These parameters are frequently employed as standard criteria for the assessment of accuracy and reliability in dCAIS and sCAIS investigations [11, 15, 20, 21].

The TA of static and dynamic navigation systems is of paramount importance, as correctly planned and placed implants minimize and avoid complications such as possible injury to essential anatomical structures, including nerves, vessels and adjacent tooth roots [11, 22, 23]. Furthermore, this treatment concept can be employed to predictably realize aspects in a manner that is consistent with patients' wishes and desired esthetic outcomes [24, 25].

Building on previous systematic reviews [26, 27, 28], this study focuses on all relevant designs—animal, in vitro, cadaver, and human—and applies strict inclusion criteria to comparative studies. A meta‐analysis evaluates the transfer accuracy of dCAIS, sCAIS, and freehand techniques in implant placement.

## 2 Methods

### 2.1 Study Protocol and Registration

Prior to undertaking this systematic review and meta‐analysis, a specific protocol was developed in accordance with the PRISMA guidelines and checklist [29, 30]. The study was registered with the International Prospective Register of Systematic Reviews (PROSPERO reference CRD42024545267 and CRD42024545259).

### 2.2 Research Question

The research questions were developed according to the PICO format; that is, the Population Intervention, Control, Outcome [31].

- PICO 1: Is there a significant difference between freehand (C) and dynamic (C, I) and static (C, I) computer‐assisted dental implant placement in terms of transfer accuracy (O) observed in cadaver and in vitro studies (P)?
- PICO 2: Is there a significant difference between freehand (C) and dynamic (C, I) and static (C, I) computer‐assisted dental implant placement in terms of transfer accuracy (O) observed in human studies (P)?
- PICO 3: Is there a significant difference between freehand (C) and dynamic (C, I) and static (C, I) computer‐assisted dental implant placement in terms of transfer accuracy (O) observed in animal studies (P)?

### 2.3 Inclusion Criteria

#### 2.3.1 General

- Comparative studies (e.g., randomized controlled trials (RCT), controlled clinical trials (CCT), case–control studies, comparative cohort studies)
- Axial, global coronal and global apical deviations should be reported as mean values with standard deviation (SD)
- Written in English or German language

#### 2.3.2 PICO 1

- At least 10 human cadaver or resin models and at least 30 implants investigated
- Fully or partially edentulous jaw models
- Dental implants with standard lengths and standard diameters

#### 2.3.3 PICO 2

- At least 10 patients and at least 30 implants investigated
- Fully or partially edentulous upper or lower jaw
- Patients 18 years of age or older with an edentulous space requiring a fixed prosthesis
- Patients who required dental implant(s) and received implant treatment
- Dental implants with standard lengths and standard diameters
- Patients with sufficient bone volume and intact extraction socket walls for implant placement

#### 2.3.4 PICO 3

- Animal studies (independent of animal species)
- At least 10 animals (5 per group)
- A minimum of one titanium dental implant (or test device) per animal

#### 2.3.5 Inclusion Thresholds

Inclusion thresholds were established to balance comprehensiveness with methodological rigor. For clinical studies (PICO 2), a minimum of 10 patients and 30 implants per study was required, in line with prior reviews evaluating accuracy in guided implant placement [15, 16]. For animal studies (PICO 3), a minimum of at least 5 animals per group with a minimum of one implant per animal was required. This is consistent with standard practices in systematic reviews of animal models in oral implantology, where such thresholds help ensure sufficient power and biological relevance [32].

### 2.4 Exclusion Criteria

#### 2.4.1 General

- Studies with unclear descriptions of the methods and results

#### 2.4.2 Specific to PICO 1/2

- Zygomatic, pterygoid, orthodontic, short or mini‐implants, test devices

#### 2.4.3 Specific to PICO 2

- Patients with contraindications for dental implant surgery including uncontrolled systemic diseases, uncontrolled diabetes and progressive periodontitis that may affect osseointegration or interfere with the healing process.

#### 2.4.4 Specific to PICO 3

- Animals with comorbidities
- Human population studies
- In‐silicio studies
- In vitro studies
- Less than required animal per group (see above)
- No dental implant/testing device implanted

For all types of designs, test and control group implant placement by using static and dynamic navigation procedures must be analyzed and reported. If there are multiple publications on the same model series or data sets, only one of the publications will be considered.

### 2.5 Search Strategy, Screening, and Study Selection

For the electronic database search predefined search terms were used (see Appendix S1). The search was performed on 31 October 2024, and an updated search was conducted on 18 June 2025. The following databases were searched electronically for peer‐reviewed publications:

- MEDLINE (Medical Literature Analysis and Retrieval System Online via PubMed)
- The Cochrane Library
- Google Scholar (to capture gray literature, the first 500 hits were selected)

A manual search for eligible publications was conducted:

- The last 10 years (based on tables of content) of the following journals: Journal of Dentistry, Clinical Oral Implants Research, Clinical Implant Dentistry and Related Research, International Journal of Computerized Dentistry, International Journal of Oral Implantology.
- Reference lists of included publications
- Experts on the topic

The initial stage of the review process involved the screening of titles and abstracts by two independent reviewers (F.R., C.R.). Abstracts that met all inclusion and exclusion criteria were considered for full‐ text review. An initial calibration exercise was undertaken, and the inter‐rater reliability was calculated as a percentage of agreement. Any disagreement was referred to a third reviewer (A.K.) for discussion and resolution. The next step was the independent full‐text search (F.R., C.R.). Any difference of opinion was referred to a third reviewer (A.K.) for the final decision.

### 2.6 Data Extraction

Two reviewers (F.S.R., C.R.) performed duplicate data extraction independently, utilizing a pre‐established and trailed spreadsheet in accordance with the recommendations of the Cochrane Handbook [31]. Based on full‐text screening of the selected studies for PICO 1 and 2, the following parameters were extracted: author(s) (year), country, number of patients (models)/implants, intervention, comparison, outcomes, drill protocol, number of operators, skill of operator(s), dentition, dynamic navigation system, measurement method and funding. Additionally, for PICO 1, the method of model fixation was reported. For PICO 2, study design, age, gender distribution and number of patients and implants were also documented in the spreadsheet.

### 2.7 Quality and Risk of Bias Assessment

To ensure transparency, completeness, and reproducibility of this work, the PRISMA guidelines and checklist (version 2020) were used (Appendix S2). Two reviewers (F.S.R., S.R.) conducted an independent assessment of the risk of bias using a range of tools. Any discrepancies were resolved by a third reviewer (A.K.).

- The Cochrane Collaboration Risk of Bias (RoB 2.0 tool) was used for randomized trials [33].
- Newcastle‐Ottawa Scale was used for observational studies [34].
- OHAT Risk of Bias Tool for Human and Animal Studies [35] was adapted to in vitro studies. This approach has been successfully used in other published systematic reviews [36].

### 2.8 Meta‐Analysis

Outcomes of TA values were analyzed using standard statistical techniques. Weighted mean difference and 95% confidence intervals were employed for continuous outcomes. The meta‐analysis, which included the determination of heterogeneity, as well as the creation of Forest Plots and Funnel Plots was conducted using RStudio, R version 4.2.3 (2023‐03‐15), (Posit PBC, USA) with the ‘meta’ package, version 6.5‐0 [37]. The data was processed for synthesis using the ‘metacont’ function for continuous data and the ‘metabin’ function for binary data, and results were presented as Forest Plots and Funnel Plots. In all cases, random effects models were utilized.

## 3 Results

A total of 4511 studies were identified through electronic and manual searches. After duplicate removal 4130 records remained. Following the screening of titles and abstracts, as well as an additional manual search based on the reference lists of the identified full texts, a total of 125 full texts were finally assessed for eligibility. 88 full texts were excluded because they did not meet the inclusion criteria. The most common justification for exclusion was non‐reporting of relevant main outcomes. A complete overview of reasons for exclusion at this stage can be found in Appendix S3.

**FIGURE 2:**
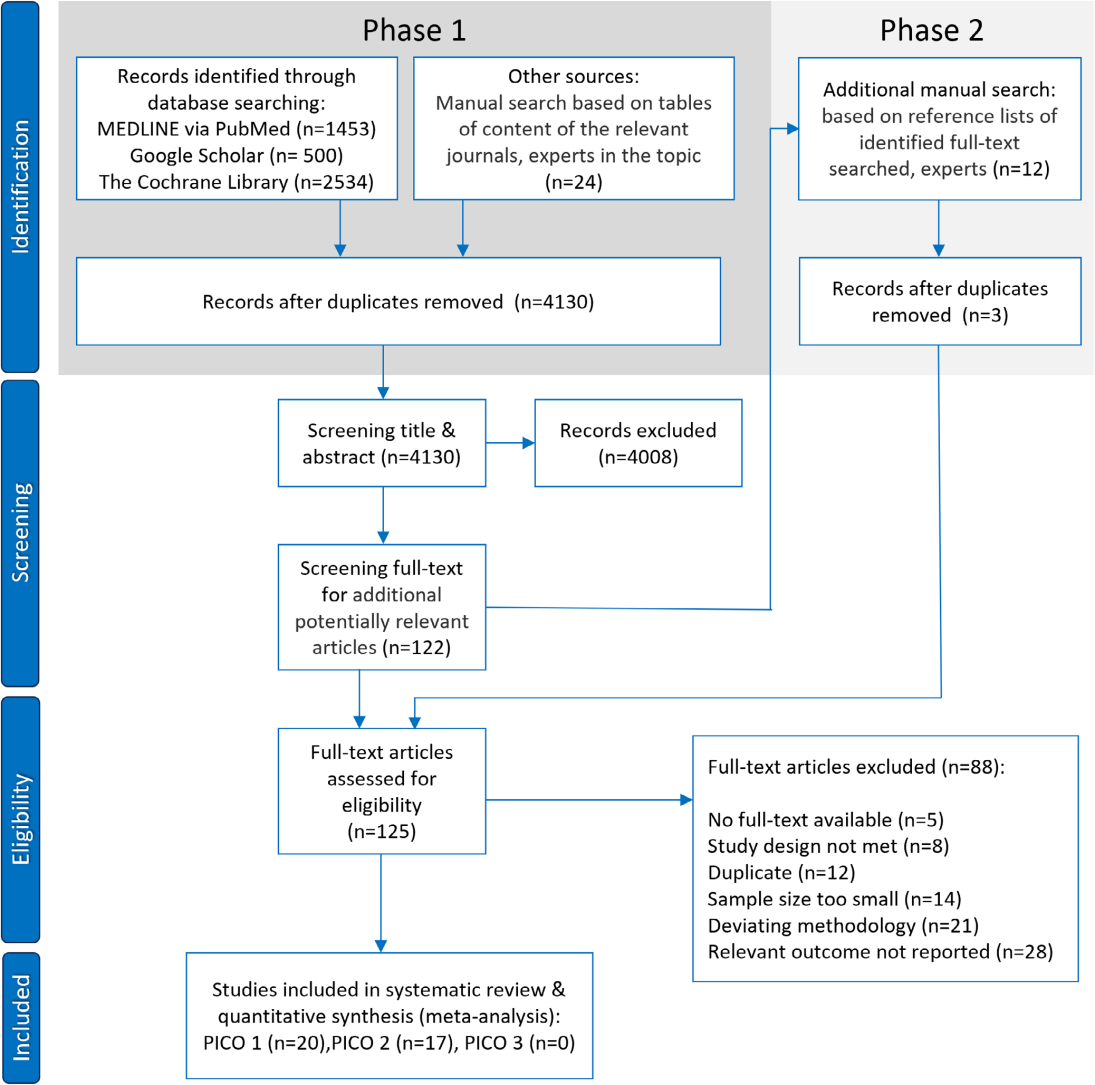
PRISMA flowchart.

A total of 37 studies were included: 20 for PICO1, 17 for PICO 2 and no studies were included for PICO 3. No eligible cadaver or animal studies were included, as none met the inclusion criteria. A flowchart for study selection is presented in Figure 2. The inter‐rater agreement at the title and abstract screening stage was 99%, with a kappa correlation coefficient of *κ* = 0.78. In accordance with the criteria set out by Landis and Koch, this indicates a substantial level of inter‐rater agreement [38].

### 3.1 PICO 1

#### 3.1.1 General

For PICO 1, data from 606 models with a total of 1869 implants were investigated in the 20 included studies. The years of publication ranged between 2019 and 2025. All eligible in vitro studies aimed to evaluate TA of freehand, dCAIS and sCAIS methods comparing presurgical implant plan and postsurgical positions.

All controlled studies compared at least two arms. Eight studies compared TA between dCAIS and sCAIS, eight compared the freehand method with sCAIS, one study compared dCAIS with freehand and three studies compared freehand, dCAIS and sCAIS. Some studies reported multiple datasets for the guided method. In this case, the means and standard deviations of the TA were pooled for the quantitative analysis [18, 39, 40, 41, 42, 43, 44, 45, 46].

In addition to the various guided methods, Abduo and Lau [40] examined the influence of long‐span edentulous areas, different implant sites and tooth supports of the templates on TA. Three studies [42, 45, 47] compared TA of implant placement in differing socket morphologies. Another study [18] investigated TA with intraoral markers and two different workflows. One study [48] investigated three different approaches: template‐guided free‐hand, sCAIS, and dCAIS and compared two different methods for determining TA. Guentsch et al. [44] examined the precision and trueness of implant placement position with and without static surgical guides and different sleeve heights.

In 12 studies the TA measurement was performed with postoperative scan bodies Abduo and Lau [39, 40, 41], Abduo et al. [49], Chen et al. [42], Guentsch et al. [44], Otaghsara et al. [50], Pitman et al. [51], Pruthi et al. [45], Shusterman et al. [52], Struwe et al. [18], and Werny et al. [53], whilst eight studies utilized post‐operative CBCTs: Fang et al. [43], Franchina et al. [48], Mampilly et al. [54], Mediavilla Guzman et al. [55], Stunkel et al. [56], Wang et al. [47], Wang et al. [46], and Zhou et al. [57]. Two further studies investigated the learning curve of inexperienced operators [46, 53].

An overview of the included studies' characteristics can be found in Table 1, and the TA results of each individual study and type of intervention are shown in Table 2. Considering the pre‐defined PICO question and the established inclusion and exclusion criteria, it was not feasible to include cadaver studies in the qualitative and quantitative analysis.

**TABLE 1:** Reported characteristics of the included studies for PICO 1.

PICO 1		P	I	C	O	Secondary aim of study	Drilling protocol	No. of operators	Skill of operators	Dentition	Model fixation	Measurement method	Dyn. navigation system	Funding
Author	Country (1st Author)	No. of models/implants	Intervention	Comparison	Outcome									
Abduo and Lau [39]	Australia	20/40 (30/60)	sCAIS	FH	Transfer accuracy	Different implant sites	sCAIS = FG	10	Inexperienced	Maxilla Partially	Phantom head	Scan body	—	The implants, surgical kits, and guide sleeves were provided by Straumann Australia. This study has been funded by the Kernot Early Career Researcher Award. No financial income for conducting the study was received by the authors
Abduo and Lau [40]	Australia	28/56 (42/84)	sCAIS	FH	Transfer accuracy	Experience, different implant sites	sCAIS = FG	14	Inexperienced	Maxilla Partially	Phantom head	Scan body	—	They would also like to thank the team of Straumann Australia for their generous support in providing the surgical kits and training implants. This study has been funded by the ITI Research Grant (1335_2018) and the Kernot Early Career Researcher Award
Abduo and Lau [41]	Australia	28/56 (42/84)	sCAIS	FH	Transfer accuracy	Different tooth support	sCAIS = FG	14	Inexperienced	Mandible Partially	Phantom head	Scan body	—	The implants, surgical kits, and guide sleeves were provided by Straumann Australia. This study has been funded by ITI Research Grant (1335_2018)
Abduo et al. [49]	Australia	30/60 (55/90)	sCAIS	FH	Transfer accuracy	Experience	sCAIS = FG	15	Inexperienced	Maxilla Partially	Phantom head	Scan body	—	This study has been funded by the ITI Research Grant (1335_2018)
Chen et al. [42]	USA	20/200 (30/300)	sCAIS	FH	Transfer accuracy	Different socket morphology	sCAIS = FG	1	Experienced	Maxilla Edentulous	NR	Scan body	—	No funding
Fang et al. [43]	USA	60/120	dCAIS	sCAIS	Transfer accuracy	Experience, different implant sites	dCAIS = FG sCAIS = FG	3	Experienced Inexperienced Nonexperienced	Maxilla Partially	Phantom head	Post CBCT	Inliant DN, Navigate Surgical Technologies Inc.	The authors would like to express their appreciation to Navigate Surgical Technologies Inc. for technical assistance and to the Center for Dental Research at Loma Linda University for financial support of this study
Franchina et al. [48]	Italy	15/90	dCAIS, sCAIS	FH	Transfer accuracy	Different measurement method	NR	3	Experienced	Mandible Partially	Model on table	Post CBCT	Navident, ClaroNav Technology	No funding
Guentsch et al. [44]	USA	80/80 (100/100)	sCAIS	FH	Transfer accuracy	Different sleeve heights	sCAIS = FG	1	NR	Mandible Partially	NR	Scan body	—	Straumann for providing the surgical instruments, implants, and components, Mr. Albrecht Schnappauf from Dentalwings for providing the treatment evaluation tool in CoDiagnostix, and Dr. Maharaj Singh from Marquette University for biostatistic support
Mampilly et al. [54]	India	20/60	dCAIS	FH	Transfer accuracy	Experience, duration, self‐confidence	dCAIS = NR	10	Inexperienced and experts	Maxilla Partially	Phantom head	Post CBCT	Navident, ClaroNav Technology	No funding
Mediavilla Guzman et al. [55]	Spain	20/40	dCAIS	sCAIS	Transfer accuracy	—	NR	NR	NR	Maxilla Partially	NR	Post CBCT	Navident, ClaroNav Technology	No funding
Otaghsara, S. et al. [50]	Switzerland	20/40	dCAIS	sCAIS	Transfer accuracy	Different implant sites	dCAIS = NR sCAIS = FG	1	Experienced	Maxilla Partially	Phantom head	Scan body	DENACAM, MiniNavident AG	Werner Siemens Foundation (MIRACLE II/mart Implants)
Pitman et al. [51]	Belgium	12/48 18/72	sCAIS	FH	Transfer accuracy	—	sCAIS = FG	6	Experienced	Maxilla Partially	NR	Scan body	—	Material support (models, implants, Scan bodies, and surgical guide sleeves) provided by Straumann Belgium
Pruthi et al. [45]	Australia	60/60 (90/90)	sCAIS	FH	Transfer accuracy	Different socket morphology	sCAIS = FG	NR	NR	Maxilla Partially	Phantom head	Scan body	—	This study was partially funded by ITI research grant and, materials for research were supplied by Straumann, Australia. Open access publishing facilitated by The University of Melbourne
Shusterman et al. [52]	Israel	45/135	dCAIS, sCAIS	FH	Transfer accuracy	—	dCAIS = FG sCAIS = FG	1	Experienced	Maxilla Partially	Model on table	Scan body	ANNA, MARS Dental	NR Author is an employee of MARS
Struwe et al. [18]	Switzerland	30/270	dCAIS	sCAIS	Transfer accuracy	Two different workflows	dCAIS = PG sCAIS = FG	2	NR	Mandible Partially	Phantom head	Scan body	DENACAM, MiniNavident AG	The study was sponsored by a funding of the Center of Dental Traumatology Basel (University Center for Dental Medicine Basel UZB). The implants were provided by Straumann AG
Stunkel et al. [56]	Germany	12/60	dCAIS	sCAIS	Transfer accuracy	Surgical time	dCAIS = PG sCAIS = PD	2	Experienced	Mandible Partially	Phantom head	Post CBCT	DENACAM, MiniNavident AG	Projekt DEAL by the Open Access Publication Funds of the Göttingen University
Wang et al. [47]	China	20/80	dCAIS	sCAIS	Transfer accuracy	Different socket morphology	NR	1	Experienced	Maxilla Partially	NR	Post CBCT	NR	No funding
Wang et al. [46]	China	30/150	dCAIS	sCAIS	Transfer accuracy	Learning curve	dCAIS = FG sCAIS = FG	3	Inexperienced (senior students)	Mandible Partially	Phantom head	Post CBCT	Iris‐100, EPED Group	Grants/awards of the Clinical Research Plan of SHDC (SHDC2020CR3049B), CAMS Innovation Fund for Medical Sciences (CIFMS) (Project No. 2019‐I2M‐5‐037) and the Research Discipline fund (No. KQYJXK2020) from Ninth People's Hospital, Shanghai
Werny et al. [53]	Germany	36/144	dCAIS, sCAIS	FH	Transfer accuracy	Learning curve, experience, surgical time	dCAIS = FG sCAIS = FG	12	Experienced and Dental students	Maxilla Partially	Phantom head	Scan body	DENACAM, MiniNavident AG	No funding
Zhou et al. [57]	China	20/80	dCAIS	sCAIS	Transfer accuracy	—	dCAIS = FG sCAIS = FG	1	NR	Mandible Partially	Phantom head	Post CBCT	Yizhimei, Digital‐health Care Medical Technology Co. Ltd.	NR

Abbreviations: FG, fully guided; FH, free hand; MR, mixed reality; NR, not reported; PD, pilot drill; PG, partially guided.

**TABLE 2:** Reported TA values of the included studies for PICO 1.

PICO 1	dCAIS						sCAIS						Freehand						dCAIS		sCAIS		Freehand	
Author	Axial deviation (°)	SD	Coronal deviation (mm)	SD	Apical deviation (mm)	SD	Axial deviation (°)	SD	Coronal deviation (mm)	SD	Apical deviation (mm)	SD	Axial deviation (°)	SD	Coronal deviation (mm)	SD	Apical deviation (mm)	SD	Models	Implants	Models	Implants	Models	Implants
Abduo and Lau [39]	—	—	—	—	—	—	2.52	1.11	0.50	0.26	0.73	0.24	4.78	2.09	1.03	0.24	0.92	1.17	—	—	10	20	10	20
Abduo and Lau [40]	—	—	—	—	—	—	1.42	0.99	0.43	0.24	0.67	0.42	5.17	3.01	1.86	0.70	2.59	1.03	—	—	14	28	14	28
Abduo and Lau [41]	—	—	—	—	—	—	1.95	1.25	0.44	0.23	0.85	0.42	5.83	3.50	1.05	0.60	1.48	0.90	—	—	14	28	14	28
Abduo et al. [49]	—	—	—	—	—	—	1.71	1.08	0.28	0.18	0.55	0.33	4.62	3.82	0.85	0.51	1.26	0.64	—	—	15	30	15	30
Chen et al. [42]	—	—	—	—	—	—	1.97	0.99	0.57	0.16	0.93	0.33	5.58	2.74	0.96	0.43	1.73	0.71	—	—	10	100	10	100
Fang et al. [43]	0.92	0.34	0.53	0.15	0.51	0.10	2.74	0.23	0.56	0.06	0.56	0.08	—	—	—	—	—	—	30	60	30	60	—	—
Franchina et al. [48]	2.76	1.38	0.89	0.37	1.31	0.68	3.23	1.00	0.79	0.35	1.17	0.48	7.41	3.87	1.65	0.61	2.33	1.01	5	30	5	30	5	30
Guentsch et al. [44]	—	—	—	—	—	—	1.54	0.57	0.37	0.13	0.56	0.17	4.86	2.10	0.80	0.35	1.53	0.74	—	—	60	60	20	20
Mampilly et al. [54]	7.16	1.76	5.34	1.45	5.89	1.08	—	—	—	—	—	—	9.06	2.18	6.19	3.14	6.95	2.12	10	30	—	—	10	30
Mediavilla Guzman et al. [55]	4.00	1.41	0.85	0.48	1.18	0.60	2.95	1.48	0.78	0.43	1.20	0.60	—	—	—	—	—	—	10	20	10	20	—	—
Otaghsara, S. et al. [50]	3.35	1.89	0.88	0.32	1.28	0.55	4.31	1.22	0.77	0.32	1.26	0.39	—	—	—	—	—	—	10	20	10	20	—	—
Pitman et al. [51]	—	—	—	—	—	—	3.05	0.97	0.81	0.21	1.47	0.31	2.60	1.41	0.59	0.50	1.13	0.54	—	—	6	24	6	24
Pruthi et al. [45]	—	—	—	—	—	—	2.51	0.41	1.31	0.19	1.79	0.47	3.79	0.67	1.62	0.35	2.40	0.73	—	—	30	30	30	30
Shusterman et al. [52]	0.85	0.32	0.33	0.16	0.40	0.16	1.20	0.39	0.36	0.12	0.49	0.12	4.43	1.72	1.08	0.41	1.53	0.66	15	45	15	45	15	45
Struwe et al. [18]	2.49	1.80	1.38	0.81	1.41	0.81	2.96	1.69	0.69	0.30	1.08	0.48	—	—	—	—	—	—	20	180	10	90	—	—
Stunkel et al. [56]	3.14	1.27	0.84	0.26	1.01	0.26	5.19	2.82	0.65	0.24	0.94	0.54	—	—	—	—	—	—	6	30	6	30	—	—
Wang et al. [47]	2.08	0.93	0.70	0.29	0.88	0.35	2.78	1.14	1.17	0.22	1.55	0.32	—	—	—	—	—	—	10	40	10	40	—	—
Wang et al. [46]	4.26	2.39	1.95	0.80	2.28	0.92	3.93	2.27	1.05	0.57	1.65	0.66	—	—	—	—	—	—	15	75	15	75	—	—
Werny et al. [53]	3.20	2.16	1.14	0.70	1.23	0.58	2.66	1.77	0.67	0.32	1.00	0.39	6.67	3.70	1.31	0.88	1.75	0.90	12	48	12	48	12	48
Zhou et al. [57]	0.97	1.21	0.40	0.41	0.34	0.33	2.60	1.11	1.15	0.34	1.37	0.38	—	—	—	—	—	—	10	40	10	40	—	—

Abbreviation: SD, Standard deviation.

#### 3.1.2 Risk of Bias

For the risk of bias analysis, the OHAT RoB‐tool was used. The results are illustrated in Table 3. The overall risk of five studies was determined to be ‘probably high’, whilst 15 studies were assessed as having a ‘probably low’ risk of bias. The higher risk assessment outcome was attributed to the domain of the randomization process, as the allocation of samples to study groups was not adequately concealed. Additionally, the studies did not account for important confounding and modifying variables. Further, there were lapses in the blinding of the operators and assessors and this was identified as a potential source of bias.

**TABLE 3:** Risk of bias assessment for PICO 1. Assessment based on the OHAT Risk of Bias (RoB) Tool, with judgment levels as follows: ++ (dark green) = definitely low risk; + (light green) = probably low risk; − − (red)= definitely high risk; − (light red) = probably high risk.

Signaling question	Abduo and Lau [39]	Abduo and Lau [40]	Abduo and Lau [41]	Abduo et al. [49]	Chen et al. [42]	Fang et al. [43]	Franchina et al. [48]	Guentsch et al. [44]	Mampilly et al. [54]	Mediavalla Guzman et al. [55]	Otaghsara et al. [50]	Pitman et al. [51]	Pruthi et al. [45]	Shusterman et al. [52]	Struwe et al. [18]	Stunkel et al. [56]	Wang et al. [47]	Wang et al. [46]	Werny et al. [53]	Zhou et al. [57]
Q 1. Was administered dose or exposure level adequately randomized?	NR	NR	NR	NR	NR	NR	+	NR	NR	++	NR	NR	NR	NR	NR	NR	+	NR	+	NR
Q 2. Was allocation to study groups adequately concealed?	NR	NR	NR	NR	NR	NR	NR	NR	NR	NR	NR	NR	NR	NR	NR	NR	NR	NR	+	NR
Q 3. Did selection of study participants result in appropriate comparison groups?	+	+	+	+	+	+	+	+	+	++	+	++	+	+	+	+	+	+	+	+
Q 4. Did the study design or analysis account for important confounding and modifying variables?	+	+	+	+	+	−	−	−	+	−	−	−	−	+	−	−	−	+	−	−
Q 5. Were experimental conditions identical across study groups?	+	+	+	+	+	+	−	+	−	−	+	+	+	−	+	+	−	−	++	+
Q 6. Were the research personnel and human subjects blinded to the study group during the study?	− −	− −	− −	− −	− −	− −	− −	− −	− −	− −	− −	− −	− −	− −	− −	− −	− −	− −	− −	− −
Q 7. Were outcome data complete without attrition or exclusion from analysis?	+	+	+	+	++	++	++	++	++	++	++	++	+	++	++	++	++	++	+	++
Q 8. Can we be confident in the exposure characterization?	+	+	+	+	+	+	+	+	+	++	+	+	+	+	+	+	+	+	+	+
Q 9. Can we be confident in the outcome assessment?	++	++	++	++	+	++	++	+	+	++	++	++	++	+	++	++	+	+	++	++
Q 10. Were all measured outcomes reported?	++	++	++	++	++	++	++	++	++	++	++	++	++	++	++	++	++	++	++	++
Q 11. Were there no other potential threats to internal validity (e.g., statistical methods were appropriate and researchers adhered to the study protocol)?	+	+	+	+	+	+	+	−	−	+	+	−	−	−	+	+	−	−	−	−
Overall risk of bias judgment	+	+	+	+	−	+	+	−	−	+	+	+	−	+	+	+	+	+	+	−

#### 3.1.3 Outcomes

Across the included studies, the majority demonstrated that both dCAIS and sCAIS techniques result in significantly higher accuracy compared to freehand placement. Several studies [39, 40, 42, 44, 45, 49, 51] consistently reported higher accuracy for sCAIS over FH, regardless of implant site, clinician experience, or socket morphology. Similarly, studies evaluating dCAIS [48, 52, 54] found it to be more accurate than FH, with some indicating comparable performance to sCAIS. When directly comparing dCAIS and sCAIS, findings were more nuanced. Several studies [18, 52, 55] concluded that both methods offer comparable accuracy. However, others [47, 57] reported significantly lower deviations with dCAIS, particularly in axial and apical parameters. Fang et al. [43] further suggested that dCAIS may be especially beneficial for less experienced clinicians due to reduced axial deviation.

Overall, the evidence supports the advantage of computer‐assisted techniques over freehand placement, with dynamic and static systems generally achieving comparable accuracy. However, dynamic systems may offer specific advantages in certain clinical scenarios, such as complex implant sites or among less experienced operators.

### 3.2 PICO 2

#### 3.2.1 General

For PICO 2, the years of publication of eligible investigations ranged between 2019 and 2025 and reported on data from 801 patients with a total of 1235 implants. Out of 17 selected investigations, 10 were RCTs, five were retrospective cohort studies, and two were retrospective case–control studies. The included studies focused on evaluating different approaches to dental implant placement, particularly comparing TA in computer‐aided implant surgery techniques. Some also incorporated immediate and delayed implant placement protocols, expanding the scope of the results. Alevizakos et al. [58], Smitkarn et al. [59], Varga Jr. et al. [60], and Verma et al. [61] investigated the differences between sCAIS and freehand implant placement. Aydemir and Arisan [62], Deng et al. [63], Yang and Geng [64], and Wu et al. [65] examined the performance of dCAIS in contrast to freehand techniques, with a focus on accuracy and clinical applicability. Several studies compared sCAIS and dCAIS directly. Feng et al. [66], Kaewsiri et al. [67], Liu et al. [68], Fu et al. [69], Yimarj et al. [70], and Okubo et al. [71] explored the accuracy of these two guided approaches under various clinical conditions including flapless procedures, primary stability, and the use of different patient trackers. Wu et al. [65] also considered the influence of surgeon experience and implant site on navigation accuracy. In addition, hybrid techniques combining static and dynamic methods were investigated by Yotpibulwong et al. [72], Younis et al. [73], and Zhang et al. [74], who aimed to assess the potential benefits of integrating both systems for enhanced accuracy and surgical control. In all investigations, a post‐CBCT was performed to analyze the TA, except in the study by Alevizakos et al. [58], who measured TA by using a scan body technique.

An overview of the studies' characteristics can be found in Table 4, and the transfer accuracy results of each individual study and distribution of patients, implants, gender, age are shown in Table 5.

**TABLE 4:** Reported characteristics of the included studies for PICO 2.

PICO 2			P	I	C	O	Secondary aim of study							
Author	Study design	Country, 1st Author	No. of Patients/Implants	Intervention	Comparison	Outcomes		Drilling protocol	No. of operators	Skill of operators	Dentition	TA Measurement method	Dyn. navigation system	Funding
Alevizakos et al. [58]	RCS	Austria	20/41	sCAIS	FH	Transfer accuracy	—	sCAIS = FG	Several	In‐experienced	Maxilla & mandible	Scan body on mastercast	—	NR
Aydemir and Arisan [62]	Split mouth RCT	Turkey	30/86	dCAIS	FH	Transfer accuracy	—	dCAIS = FG	1	Experienced	Maxilla Partial edentulous	Post CBCT	Navident, ClaroNav	This study was supported by the grants of Istanbul University Research Fund (TSA 8331–1935, BYP 2018–30 120 and 509–29 870).
Deng et al. [63]	RCS	China	55/72	dCAIS	FH	Transfer accuracy	Clinical effect of immediate implant	dCAIS = FG	NR	NR	Maxilla	Post CBCT	Iris‐100, EPED Group	No funding
Feng et al. [66]	RCT	China	40/40	dCAIS	sCAIS	Transfer accuracy	—	dCAIS = FG sCAIS = FG	1	Experienced	Maxilla—partially	Post CBCT	Dcarer, Suzhou Digital‐health care Co. Ltd.	Crosswise Project (Grant number 00403055A1091)
Fu et al. [69]	RCS	China	22/110	dCAIS	sCAIS	Transfer accuracy	PROMS, flapless, time	dCAIS = FG sCAIS = FG	2	NR	Maxilla & mandible, fully edentulous	Post CBCT	Yizhimei, Digital‐health Care Medical Technology Co. Ltd.	This study was supported by the Wuhan University Specific Fund for Major School‐level Internationalization Initiatives (WHUGJZDZX‐RC07) and an ITI research grant (1721–2022).
Kaewsiri et al. [67]	RCT	Thailand	60/60	dCAIS	sCAIS	Transfer accuracy	Flapless, time	dCAIS = FG sCAIS = FG	1	Experienced	Maxilla & mandible—NR	Post CBCT	NR	Supported by the 90th Anniversary of Chulalongkorn University Rachadapisek Sompote Fund.
Liu et al. [68]	RCS	China	32/38	dCAIS	sCAIS	Transfer accuracy	Primary stability, osteotomy accuracy	dCAIS = FG sCAIS = FG	2	Experienced	Maxilla—partially	Post CBCT	DHC DI2; Dcarer, Suzhou Digital‐health care Co. Ltd.	NR
Okubo et al. [71]	RCS	Japan	42/50	dCAIS	sCAIS	Transfer accuracy	Patient tracker surgical guide	dCAIS = FG sCAIS = FG	3	NR	Partially edentulous	Post CBCT	X‐guide (X‐Nav Technologies)	No funding
Smitkarn et al. [59]	RCT	Thailand	52/60	sCAIS	FH	Transfer accuracy	—	sCAIS = FG	1	Experienced	Maxilla & mandible, partially	Post CBCT	—	This research was supported by the 90th Anniversary of Chulalongkorn University Rachadapisek Sompote Fund.
Varga Jr. et al. [60]	RCT	Hungary	54/107 (101/207)	sCAIS	FH	Transfer accuracy	—	sCAIS = FG	Several	NR	Maxilla & mandible	Post CBCT	—	Endre Varga is the CEO of dicomLAB Dental Ltd.; Gábor Braunitzer is the chief researcher of dicomLAB Dental Ltd.
Verma et al. [61]	Split mouth RCT	India	20/40	sCAIS	FH	Transfer accuracy	—	sCAIS = NR	NR	NR	Maxilla & mandible Partial edentulous	Post CBCT	—	No funding
Wu et al. [65]	RCS	China	54/95	dCAIS	sCAIS	Transfer accuracy	Experience of the surgeon, different implant sites	dCAIS = FG sCAIS = FG	3	2 experienced, 1 novice	NR	Post CBCT	DHC‐DI3E, Suzhou Digital‐health care Co. Ltd.	Natural Science Foundation of Fujian Province [grant numbers: 2018J0106] and the Science Foundation of Fujian Province [grant numbers: 2019J01319]
Yang and Geng [64]	RCT	China	60/96	dCAIS	FH	Transfer accuracy	Clinical effect of immediate implant	dCAIS = FG	1	NR	Maxilla	Post CBCT	Iris‐100, EPED Group	No funding
Yimarj et al. [70]	RCT	Thailand	30/60	dCAIS	sCAIS	Transfer accuracy	Parallelism	dCAIS = FG sCAIS = FG	1	Experienced	NR partially	Post CBCT	NR	NR
Yotpibulwong et al. [72]	RCT	Thailand	90/90 (120/120)	dCAIS sCAIS	FH	Transfer accuracy	Double factor (combination of dCAIS with sCAIS)	dCAIS = FG sCAIS = FG	1	Experienced	Maxilla & mandible, partially	Post CBCT	Iris‐100, EPED Group	Supported by the 90th Anniversary of Chulalongkorn University Rachadapisek Sompote Fund.
Younis et al. [73]	RCT	China	65/94	dCAIS, sCAIS	FH	Transfer accuracy	Advantages and disadvantages	dCAIS = NR sCAIS = NR	1	Experienced	Maxilla & mandible, partially & fully edentulous	Post CBCT	Dcarer, Suzhou Digital‐health care Co. Ltd.	This work was supported by the National Natural Science Foundation of China (22078209) to W. L. and the Medical Research Project of Xi'an Science and Technology Bureau of China‐General Project (2022JH‐YBYJ‐0354) to L.H.
Zhang et al. [74]	RCS	China	75/96	dCAIS sCAIS	FH	Transfer accuracy	—	dCAIS = FG sCAIS = FG	Several	Experienced	Mandible	Post CBCT	Iris‐100, EPED Group	This research is supported by the Natural Science Foundation of Guangdong Province, China (Grant No. 2022A1515010809)

Abbreviations: FG, fully guided; FH, free hand; NR, not reported; RCS, retrospective cohort study; RCT, randomized controlled study.

**TABLE 5:** Reported TA values and distribution of patients, implants, gender and age for PICO 2.

PICO 2	dCAIS						sCAIS						Freehand						dCAIS (distribution, gender, age)					sCAIS (distribution, gender, age)					Freehand (distribution, gender, age)				
Author	Axial deviation (°)	SD	Coronal deviation (mm)	SD	Apical deviation (mm)	SD	Axial deviation (°)	SD	Coronal deviation (mm)	SD	Apical deviation (mm)	SD	Axial deviation (°)	SD	Coronal deviation (mm)	SD	Apical deviation (mm)	SD	Patients	Implants	Male	Female	Age range Ø age	Patients	Implants	Male	Female	Age range Ø age	Patients	Implants	Male	Female	Age range Ø age
Alevizakos et al. [58]	—	—	—	—	—	—	3.4	1.7	1.26	0.68	1.47	0.67	9.70	4.40	2.12	1.28	2.92	1.91	—	—	—	—	—	8	20	4	4	56.2	12	21	5	7	57.1
Aydemir and Arisan [62]	5.59	0.39	1.01	0.07	1.83	0.12	—	—	—	—	—	—	10.40	0.83	1.70	0.13	2.51	0.21	30	43	NR	NR	NR	—	—	—	—	—	30	43	NR	NR	NR
Deng et al. [63]	1.45	0.39	0.43	0.15	0.77	0.28	—	—	—	—	—	—	3.05	1.17	1.35	0.58	1.55	1.03	29	38	18	11	41.7 ± 16.78	—	—	—	—	—	26	34	12	14	47.26 ± 19.83
Feng et al. [66]	3.23	1.67	1.06	0.55	1.18	0.53	3.07	2.18	0.99	0.63	1.5	0.75	—	—	—	—	—	—	20	20	9	11	36.4 ± 13.11	20	20	9	11	42.6 ± 12.83	—	—	—	—	—
Fu et al. [69]	3.87	2.75	1.02	0.45	1	0.51	2.32	1.23	1.56	1.19	1.7	1.09	—	—	—	—	—	—	11	54	7	4	≤ 40 = 4 < 40 ≤ 60 = 5 > 60 = 2 range: 20–75	11	56	7	4	≤ 40 = 0 < 40 ≤ 60 = 4 > 60 = 7 range: 20–75	—	—	—	—	—
Kaewsiri et al. [67]	3.06	1.37	1.05	0.44	1.29	0.5	2.84	1.71	0.97	0.44	1.28	0.46	—	—	—	—	—	—	30	30	7	23	50 range: 21–70	30	30	9	21	57 range: 28–74	—	—	—	—	—
Liu et al. [68]	2.14	1.2	1.07	0.57	1.26	0.53	3.31	1.61	0.92	0.46	1.31	0.43	—	—	—	—	—	—	16	20	9	7	41 ± 13.73	16	18	4	12	38.38 ± 10.51	—	—	—	—	—
Okubo et al. [71]	2.64	0.87	0.99	0.33	0.97	0.43	3.42	1.03	1.33	0.26	1.38	0.30	—	—	—	—	—	—	21	25	NR	NR	NR	21	25	NR	NR	NR	—	—	—	—	—
Smitkarn et al. [59]	—	—	—	—	—	—	3.1	2.3	1	0.6	1.3	0.6	6.90	4.40	1.50	0.70	2.10	1.00	—	—	—	—	—	26	30	NR	NR	NR	26	30	NR	NR	NR
Varga Jr. et al. [60]	—	—	—	—	—	—	3.04	1.51	1.4	0.54	1.59	0.59	7.03	3.44	1.82	0.94	2.43	0.98	—	—	—	—	—	28	52	15	13	42.11	26	55	13	13	40.38
Verma et al. [61]	—	—	—	—	—	—	14.63	8.69	2.16	0.92	4.08	1.88	11.89	7.53	2.00	1.13	3.52	1.66	—	—	—	—	—	20	20	NR	NR	NR	20	20	NR	NR	NR
Wu et al. [65]	3.71	1.32	1.36	0.65	1.48	0.65	4.34	2.22	1.22	0.7	1.33	0.73	—	—	—	—	—	—	25	38	NR	NR	37 ± 16.7 range: 19–67	29	57	NR	NR	37 ± 16.7 range: 19–67	—	—	—	—	—
Yang and Geng [64]	1.03	0.55	0.56	0.07	0.49	0.26	—	—	—	—	—	—	3.35	1.12	1.26	0.13	1.33	0.42	32	50	?	?	43.7 ± 17.59	—	—	—	—	—	28	46	?	?	48.26 ± 20.15
Yimarj et al. [70]	3.78	1.84	1.24	0.39	1.58	0.56	4.08	1.69	1.04	0.67	1.54	0.79	—	—	—	—	—	—	15	30	5	10	60	15	30	2	13	60	—	—	—	—	—
Yotpibulwong et al. [72]	3.28	1.57	1.02	0.45	1.28	0.5	3.18	2.04	1.06	0.67	1.4	0.71	7.5	4.06	1.48	0.68	2.18	0.95	30	30	14	16	51 range: 21–70	30	30	13	17	59 range: 28–78	30	30	18	12	58 range; 27–76
Younis et al. [73]	3.66	1.64	0.99	0.52	1.14	0.56	2.52	1.18	0.92	0.36	1.06	0.47	5.82	2.79	1.36	0.62	1.73	0.66	19	34	11	8	45.9 range: 24–73	23	30	14	9	40.8 range: 22–69	23	30	8	15	44.8 range: 24–61
Zhang et al. [74]	0.89	0.58	0.24	0.16	0.47	0.32	1.87	0.53	0.26	0.15	1.46	0.36	7.52	2.44	4.26	1.36	5.84	3.11	25	32	14	11	40.1 ± 98.4	27	34	15	12	45.53 ± 12.02	23	30	13	10	41.23 ± 11.96

Abbreviations: NR, not reported; SD, standard deviation.

#### 3.2.2 Risk of Bias

For risk of bias evaluation, the NOS tool was used for five non‐randomized cohort studies and two case–control studies. The overall quality of the articles was acceptable; the case–control study was rated as 4 and 5 points (moderate risk of bias), the cohort studies were rated as 7 and 9 points (low risk of bias), illustrated in Table 6. The risk of bias of the ten included RCTs was assessed with the Cochrane risk‐of‐bias tool 2.0. Four articles were classified as having a low risk of bias, while four were considered to have some concerns and two studies were rated with high risk of bias, specifically regarding the lack of randomization process, adherence to the concealment and blinding rules. The results of the RCTs were visualized via ROBVIS [75], presented in Table 6.

**TABLE 6:** Risk of bias assessment for PICO 2, presenting evaluations using two tools: (A) RoB 2 for randomized controlled trials (RCTs), and (B) the Newcastle‐Ottawa Scale (NOS) for non‐randomized studies.

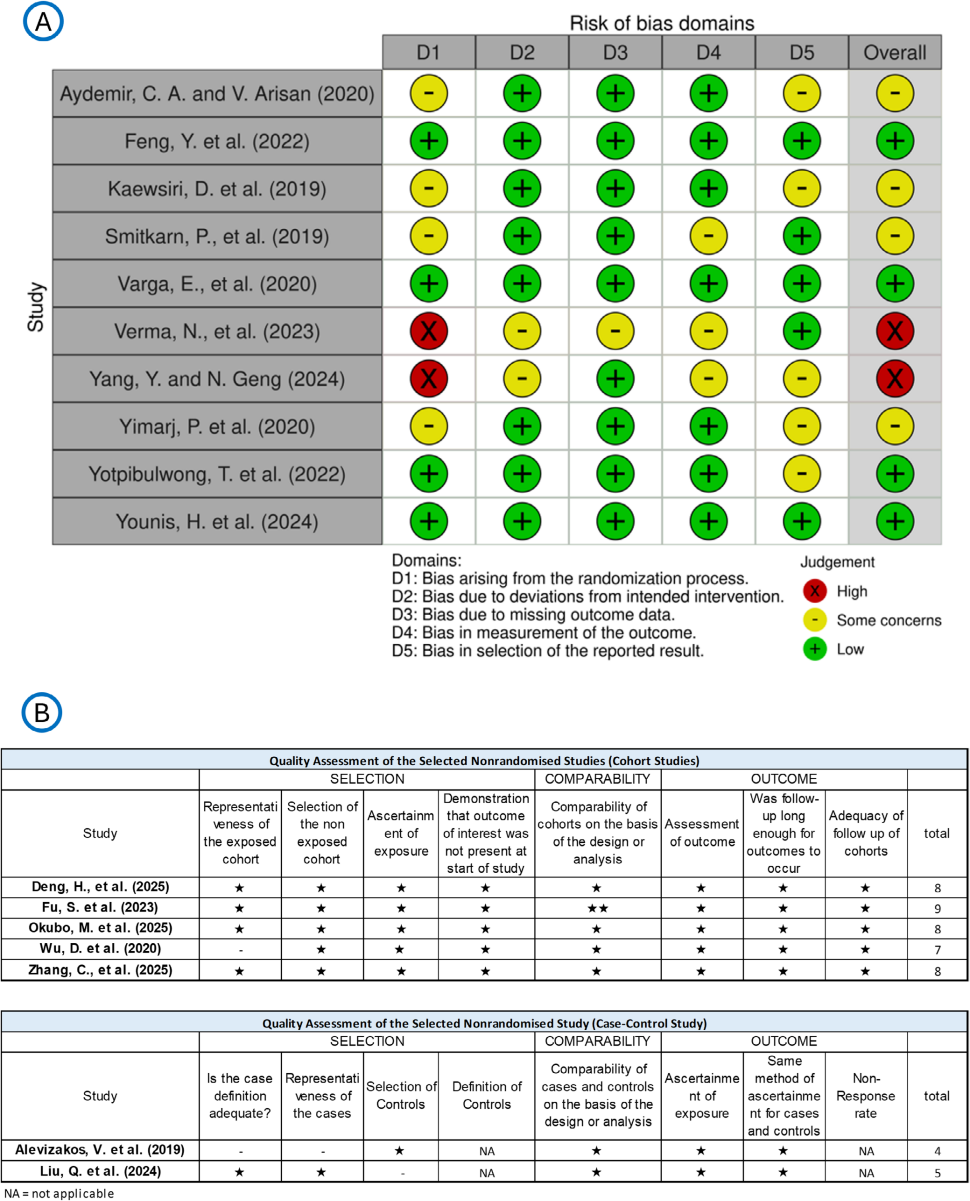

Abbreviation: NA, not applicable.

#### 3.2.3 Outcome

Numerous studies have investigated the accuracy and clinical outcomes of computer‐aided implant surgery, comparing static (sCAIS), dynamic (dCAIS), and freehand (FH) techniques. Alevizakos et al. [58] and Smitkarn et al. [59] demonstrated that sCAIS significantly improves implant placement accuracy compared to freehand, particularly for less experienced clinicians. Similarly, Varga Jr. et al. [60] confirmed the better accuracy of static guidance over conventional methods, while Verma et al. [61] found comparable accuracy between sCAIS and traditional techniques. Dynamic navigation has shown promising results in several studies. Aydemir and Arisan [62] reported that dCAIS effectively transfers virtual planning to the clinical setting with enhanced accuracy. Deng et al. [63] observed significantly reduced deviations in implant positioning with dCAIS compared to freehand, and Yang and Geng [64] confirmed these findings situated in the maxillary posterior region. Wu et al. [65] noted that dCAIS accuracy is unaffected by surgeon experience or implant site, outperforming sCAIS in molar regions. Comparative studies between dCAIS and sCAIS revealed mixed findings. Feng et al. [66] and Yimarj et al. [70] concluded that both systems achieve clinically acceptable accuracy and similar implant parallelism. Kaewsiri et al. [67] found no significant difference in flapless single‐tooth procedures. However, Liu et al. [68] reported higher axial deviation with sCAIS but greater primary stability due to higher osteotomy accuracy. Fu et al. [69] emphasized the reliability of dCAIS in fully edentulous patients, with reduced linear deviation. Okubo et al. [71] suggested that oral appliance‐based dCAIS offers greater accuracy than static guides. Hybrid approaches have also been explored. Yotpibulwong et al. [72] demonstrated that combining dCAIS and sCAIS yields significantly higher accuracy than either method alone or freehand. Younis et al. [73] highlighted the higher accuracy and consistency of both CAIS techniques over freehand, with dCAIS offering added flexibility. Finally, Zhang et al. [74] confirmed that flapless dCAIS enhances surgical safety and visualization precision.

### 3.3 PICO 3

No animal studies were found and included in the qualitative and quantitative analysis for the research question PICO 3.

### 3.4 Meta‐Analyses

#### 3.4.1 Meta‐Analysis of PICO 1

The conducted meta‐analyses comparing freehand implant placement accuracy to sCAIS and dCAIS consistently demonstrated that both latter were statistically significantly more accurate across all analyzed deviation parameters (Figure S1).

**FIGURE 3:**
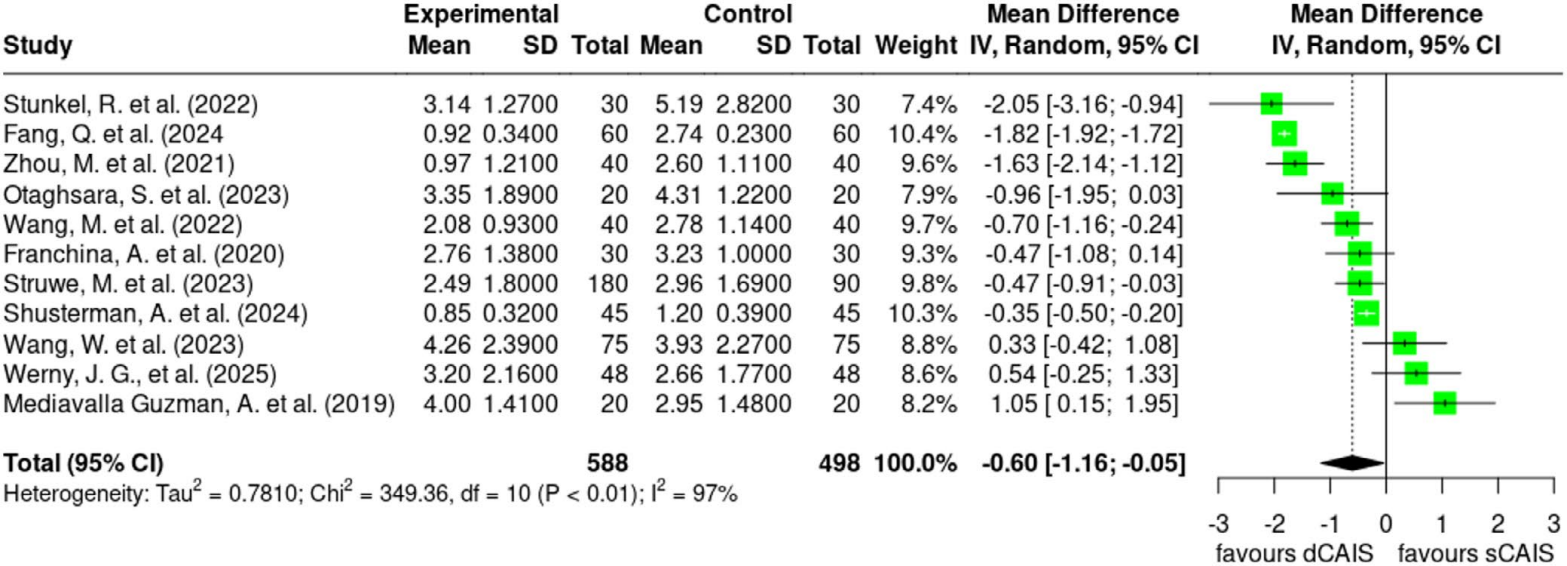
Forest plot demonstrates the analyzed transfer accuracy for PICO 1 comparison of dCAIS vs. sCAIS in axial deviation (degrees).

Comparing dCAIS with sCAIS, a statistically significant difference was observed only for the axial deviation parameter with an overall higher accuracy in dCAIS, presented in Figure 3. The pooled mean difference was −0.60 [95% CI: −1.16 to −0.05], indicating that, on average, subjects in the experimental dCAIS groups showed significantly lower deviation compared to those in the control sCAIS groups. The results of several studies statistically significantly favored dCAIS in terms of accuracy including Stunkel et al. [56] with a mean difference of −2.05° [95% CI: −3.16 to −0.94], Fang et al. [43] with −1.82° [95% CI: −1.92 to −1.72] and Zhou et al. [57] with −1.63° [95% CI: −2.14 to −1.12]. Other studies in this group showed no statistical significance, while one investigation demonstrated a clear statistically significant difference in favor of sCAIS, with a mean difference of 1.05° [95% CI: 0.15 to −1.95] [55]. The substantial variation in effect sizes and statistical significance indicates a high level of heterogeneity, as reflected by an *I* ^2^ value of 97%. This is likely attributable to differences in study design, sample characteristics, or intervention protocols. In summary, the results indicate a statistically significant difference in transfer accuracy favoring dCAIS, although the observed heterogeneity should be considered when interpreting the findings.

In the comparison between dCAIS and sCAIS, the meta‐analysis revealed no statistically significant differences in global coronal or global apical deviation, illustrated in Figure S1. Further, related funnel plots reveal high heterogeneity (Figure S2).

#### 3.4.2 Meta‐Analysis of PICO 2

Aligned with the PICO 1 findings in the previous paragraph, the meta‐analyses of the included datasets for PICO 2 revealed significantly higher accuracy in both, dCAIS and sCAIS, compared to the freehand method, presented in Figures S3 and S4.

**FIGURE 4:**
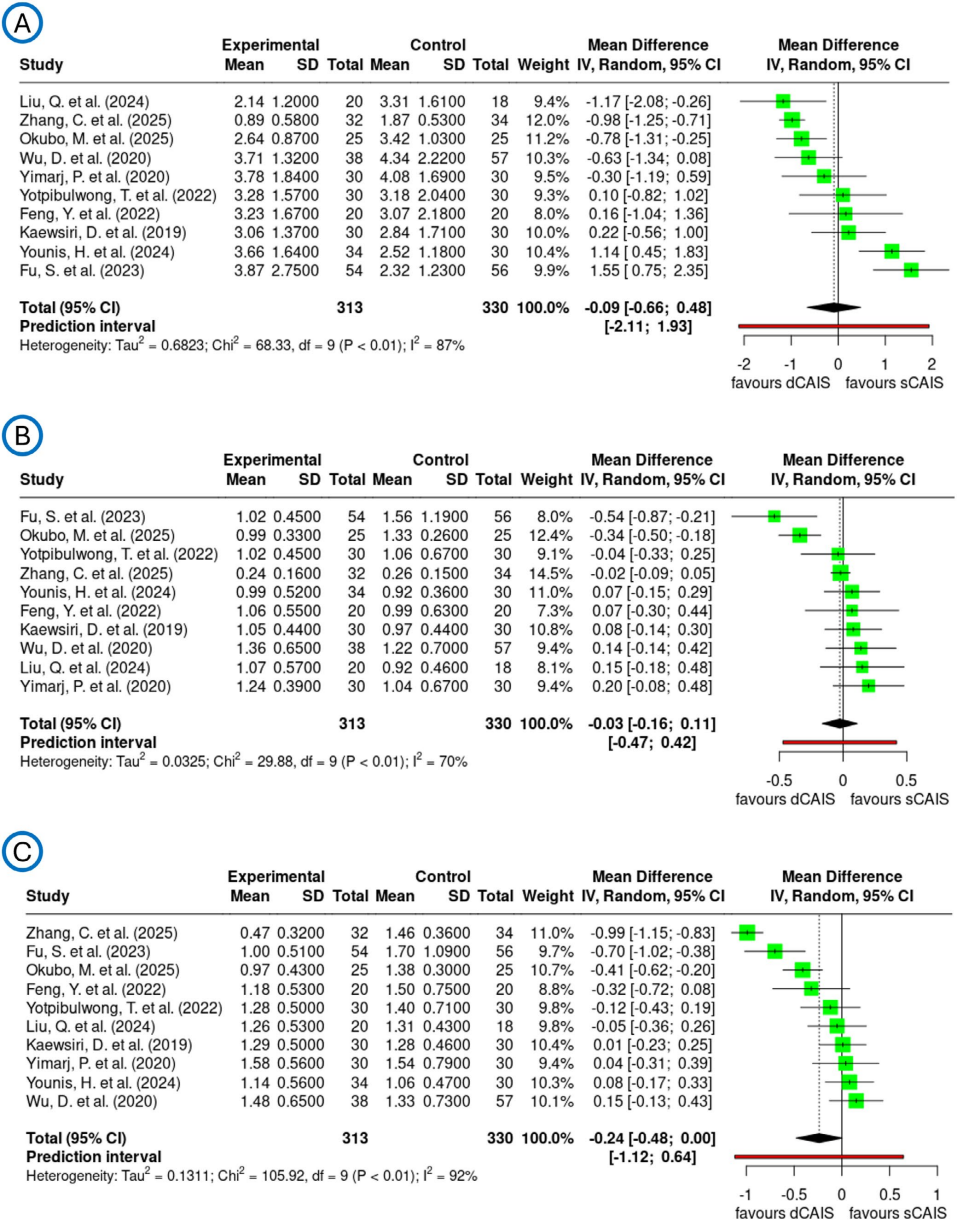
Forest plots demonstrate the analyzed transfer accuracy for PICO 2 comparison of dCAIS vs. sCAIS across the three deviation parameters: (A) axial deviation in degrees, (B) global coronal deviation in millimeters, and (C) global apical deviation in millimeters.

In the comparison between dCAIS and sCAIS, deviation parameters were analyzed through separate meta‐analyses, illustrated in Figure 4. For axial deviation, the pooled mean difference was −0.09 degrees with a 95% confidence interval ranging from −0.66 to 0.48 degrees. The corresponding 95% prediction interval ranged from −2.11 to 1.93, indicating that the true effect in a new, comparable study could fall within this broad range and indicating no statistically significant difference between the two techniques. However, the heterogeneity was substantial, with an *I* ^2^ value of 87%. Similarly, the global coronal deviation showed a mean difference of −0.03 mm [95% CI: −0.16 to 0.11] with a PI of [−0.47; 0.42], indicating no significant difference in accuracy. The heterogeneity for this parameter was moderate, with an *I* ^2^ of 70%, reflecting some inconsistency across studies. For global apical deviation, the pooled mean difference was −0.24 mm with a 95% confidence interval of −0.48 to 0.00 with a PI of [−1.12; 0.64], reflecting a slight trend in favor of dCAIS. Notably, this parameter showed considerable heterogeneity, with an *I* ^2^ value of 92%. Heterogeneities were confirmed by funnel plots, which revealed large variation demonstrated in Figure S5.

**FIGURE 5:**
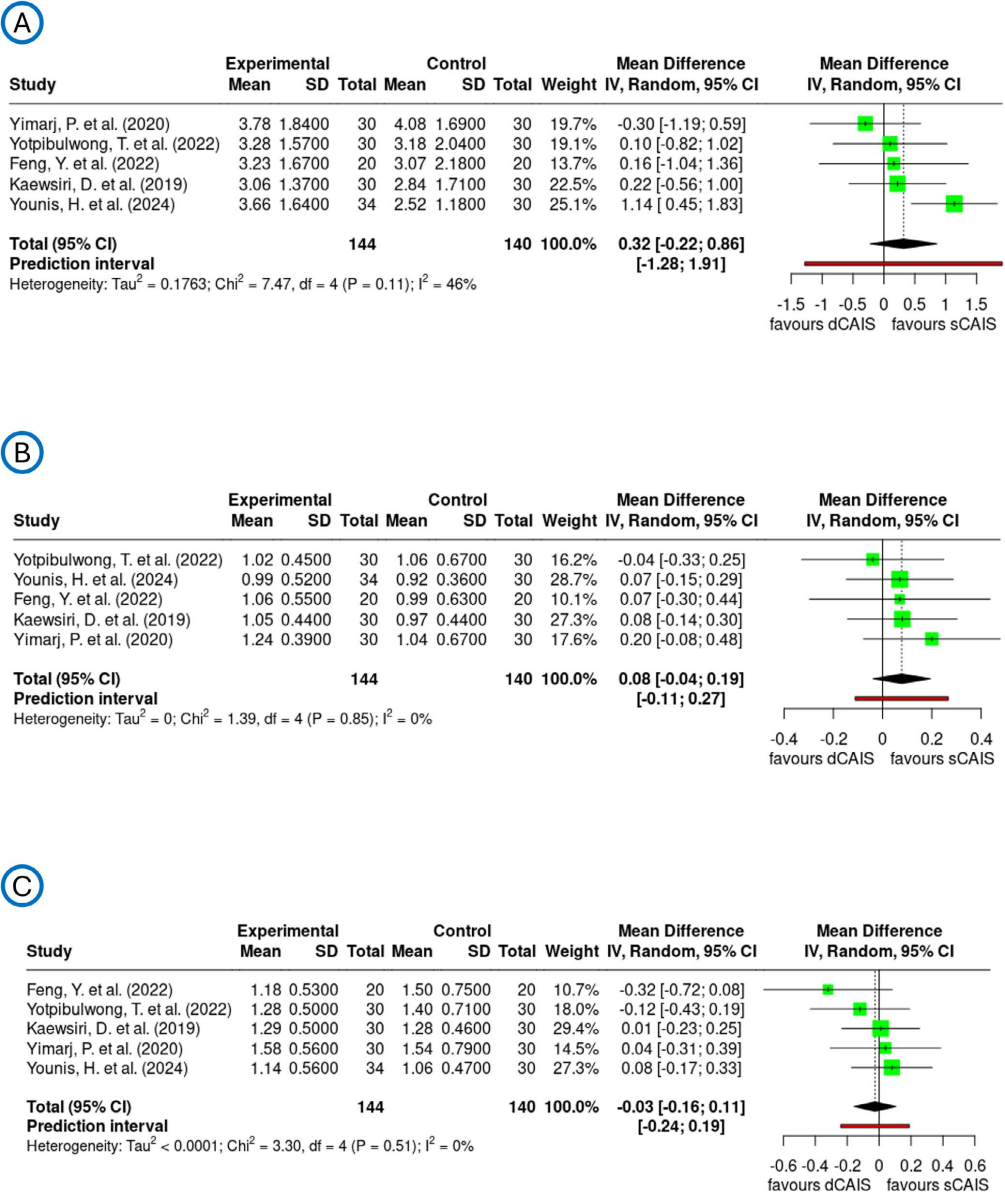
Forest plots demonstrate the analyzed transfer accuracy for PICO 2 with RCT studies only and comparison of dCAIS vs. sCAIS across the three deviation parameters: (A) axial deviation in degrees, (B) global coronal deviation in millimeters, and (C) global apical deviation in millimeters.

To reduce heterogeneity, an additional subgroup meta‐analysis focusing on the RCT datasets was conducted (Figure 5). Here, the pooled mean difference in axial deviation was 0.32 degrees with a 95% confidence interval ranging from −0.22 to 0.86 degrees and a PI of −1.28 to 1.91 degrees, indicating no statistically significant difference between the two techniques. The heterogeneity for this parameter was moderate, with a noted lower *I* ^2^ value of 46% compared to previous analyses. For global coronal deviation, the pooled mean difference was 0.08 mm [95% CI: −0.04 to 0.19; PI: −0.11; 0.27], similarly showing no significant difference between dCAIS and sCAIS. The heterogeneity was low, with an *I* ^2^ of 0%, indicating high consistency across the included studies. The analysis of global apical deviation revealed a pooled mean difference of −0.03 mm [95% CI: −0.16 to 0.11; PI: −0.24; 0.19]. The heterogeneity for this parameter was low, with an *I* ^2^ value of 0%, reflecting high homogeneity among the studies. These findings suggest that axial, coronal and apical deviations do not differ significantly between dCAIS and sCAIS. The funnel plots support these findings and suggest a low risk of publication bias (Figure S6).

## 4 Discussion

This study aimed to assess the transfer accuracy of freehand, static (sCAIS), and dynamic (dCAIS) implant placement methods. While comparisons with freehand techniques are included for completeness [15, 16, 26, 27, 28], the primary focus lies on the evaluation of sCAIS versus dCAIS. This systematic review and meta‐analysis offers new insights by including recent high‐quality RCTs and studies across all relevant designs (animal, in vitro, cadaver, human) up to June 2025. Strict inclusion criteria ensured large sample sizes, standardized methods, rigorous bias assessment, and exclusively comparative studies. Outcomes were harmonized, with transfer accuracy measured using consistent metrics.

The results of the clinical studies demonstrated that the deviations observed in the dCAIS group were not significantly different from those in the sCAIS group at global coronal (−0.03 mm [95% CI: −0.16 to 0.11]), global apical (−0.24 mm [95% CI: −0.48 to 0.00]) and axial deviation (−0.09° [95% CI: −0.66 to 0.48]). However, due to the substantial heterogeneity in the clinical trial population, with *I* ^2^ values of 70% for global coronal, 92% for global apical and 87% for axial measurements, a subgroup consisting solely of RCTs revealed significant improvements: both global coronal and global apical measurements showed an *I* ^2^ value of 0%, while the axial *I* ^2^ value decreased to 46%. The subgroup analysis exhibits similar tendencies as the overall PICO 2 analysis with pooled mean in axial 0.32° [95% CI: −0.22 to 0.86], global coronal 0.08 mm [95% CI: −0.04 to 0.19] and global apical −0.03 mm [95% CI: −0.16 to 0.11], but showed substantially lower heterogeneity and possible publication bias across all deviation parameters (Figure 5). The formation of the subgroup exclusively comprising RCTs likely enhances the observed homogeneity, as RCTs follow highly standardized protocols and exhibit greater methodological uniformity. For instance, all included RCTs applied the same drilling protocol (fully guided), and a uniform accuracy assessment method using postoperative CBCT was employed. Additionally, implant placement was performed by a single experienced surgeon in nearly all cases. The low heterogeneity of the results supports the robustness of the meta‐analysis findings; however, the results should still be interpreted with caution due to the limited number of included studies.

The mean differences across in vitro studies demonstrated that implants placed with dCAIS exhibited comparable deviation values to the sCAIS approach at global coronal (0.11 mm [95% CI: −0.17 to 0.39]) and global apical (−0.05 mm [95% CI: −0.32 to 0.23]) measurement sites. However, the axial deviations were significantly (*p* < 0.01) smaller with dCAIS (−0.60° [95% CI: −1.16 to −0.05]). Due to their controlled, model‐based design, in vitro studies may not fully reflect clinical conditions. Consequently, they often report lower deviations in transfer accuracy compared to clinical studies [15].

Errors affecting transfer accuracy in both dCAIS and sCAIS may occur during preoperative diagnostics and planning [21, 76]. Inaccuracies in radiographs can occur due to a variety of equipment quality and type [77], but also due to the patient's movements during the CBCT scan [78, 79]. Inaccuracies may also occur during the transfer of imaging data sets to the planning software, which may affect TA [80, 81]. Regarding the sCAIS method, navigation systems from different manufacturers can influence TA outcomes [82, 83], as can the choice of drilling protocol [84, 85]. The clinical procedure must take the individual anatomical characteristics, such as bone density and sufficient bone volume, into account [86]. But also the patient's cooperation, in particular muscle tension in the lips or cheek during the surgery may be relevant [87], as well as limited view of the surgical field, for example due to blood and saliva [88]. Even the expertise and level of practical experience could affect the results of the transfer accuracy [89], whereas experienced operators achieved better results in terms of accuracy [90]. For dCAIS, the choice of the respective drilling protocol used [91, 92] appears to have an influence on TA. Different registration techniques in imaging [18, 93, 94] and calibration method of the handpiece with the triangulation system [95] could also affect TA values. Expertise, in particular the learning curve experienced by the operators after repeated use of dCAIS, has been investigated in several publications as a possible influencing factor [96, 97, 98].

The literature outlines a variety of advantages and disadvantages of both dCAIS and sCAIS, which ought to be considered. Dynamic navigation systems provide surgeons real‐time feedback on the position and angle of the drill, enabling corrections to be made during the procedure to place the implant in the optimal position [64]. Furthermore, dCAIS enables surgeons to maintain an unobstructed view of the surgical site for accurate interventions, while sCAIS and its surgical guide may obstruct the surgeon's view [47, 48, 99]. Despite these potential benefits described in the literature, there are also some drawbacks regarding dCAIS mentioned, including the constant need for the surgeon to change the view between the surgical field and the monitor [55]. However, augmented reality devices may be used in the future to display the virtual image of the computer‐aided dynamic navigation system without losing sight of the surgical field [100]. A longer duration of the surgery due to the drill registration at dCAIS was mentioned as a drawback [47, 67].

### 4.1 Limitations

This review highlights considerable heterogeneity across the included studies, particularly in regard to study design, sample sizes, surgical protocols, operator experience, measurement methods, and navigation systems. These variations limited the ability to fully account for confounding influences and likely contributed to the variability in reported outcomes. The risk of bias was most pronounced in in vitro studies, where randomization and blinding were often insufficient. Most clinical studies assessed transfer accuracy with postoperative CBCT, which, while practical and widely used, is prone to scatter artifacts and introduces additional radiation exposure. Although surface scan‐based methods have been shown to be comparable or even more precise in accuracy [48, 101, 102], they remain less commonly utilized. In addition, many of the included studies were small, and long‐term data on outcomes such as implant survival and peri‐implant complications are missing. These limitations underline the need for future research with larger cohorts, harmonized protocols, validated measurement techniques, and systematic reporting of patient‐centered outcomes.

### 4.2 Clinical Considerations

#### 4.2.1 Transfer Accuracy and Surgical Time

Both static and dynamic CAIS achieve higher accuracy than freehand placement. However, efficiency is a relevant clinical factor. Several studies have reported longer procedure times with dynamic navigation, particularly due to screen‐viewing shifts or registration steps during the learning phase [46, 47, 55, 56]. More recent work indicates that workflow adaptations, including mixed‐reality approaches, may mitigate these drawbacks [18, 52].

#### 4.2.2 Cost‐Effectiveness

Economic considerations are also important. CAIS protocols generally require greater planning, and chairside time, which increases costs compared to conventional workflows [103]. While the accuracy benefits of sCAIS and dCAIS are evident, cost‐effectiveness appears highly dependent on case complexity, operator experience, and clinical setting.

#### 4.2.3 Clinical Complications

Despite improved placement accuracy, current evidence does not consistently show reductions in marginal bone loss, peri‐implant complications, or postoperative pain when using CAIS compared with conventional methods [104, 105]. This suggests that transfer accuracy alone may not guarantee superior long‐term clinical outcomes. Consequently, the choice between sCAIS and dCAIS should balance accuracy gains with surgical efficiency, economic feasibility, and the specific demands of the clinical scenario.

## 5 Conclusion

The reviewed studies consistently demonstrate that both dCAIS and sCAIS offer significantly higher transfer accuracy compared to the freehand method. In vitro studies demonstrated comparable outcomes for global coronal and global apical deviations in terms of transfer accuracy. However, in dCAIS accuracy was found to be statistically significantly higher than sCAIS for axial deviation, but the degree is of questionable clinical relevance. The results across included clinical studies demonstrate no statistically significant differences between dCAIS and sCAIS. The combination of both dynamic navigation with surgical guides may lead to less deviation.

### 5.1 Implications for Clinical Practice

The findings suggest that both dynamic (dCAIS) and static (sCAIS) computer‐assisted implant surgeries offer comparable transfer accuracy in clinical settings and exhibit higher accuracy compared to the freehand method. Clinicians can confidently use either method, understanding that computer‐assisted implantation protocols offer higher accuracy compared to freehand methods, as well as dynamic navigation may offer slight advantages in axial deviation. However, given the limited clinical relevance of the difference in axial deviation in clinical studies, the choice of system used should be guided by factors such as clinician preference, experience, time, cost, available technology, specific case and anatomical requirements. The combination of dynamic navigation with surgical guides may enhance accuracy and reduce deviation further. To assess potential safety margins during preoperative planning, the upper limits of the confidence intervals from the current meta‐analysis were considered. Based on these data, approximate values for computer‐assisted implant placement may serve as preliminary references for risk assessment: 1.4 mm (dCAIS) and 1.6 mm (sCAIS) in the coronal plane, 1.6 mm (dCAIS) and 1.7 mm (sCAIS) in the apical plane, and angular deviations of 3.9° (dCAIS) and 4.3° (sCAIS). These values are based on statistical data and should not be interpreted as clinical recommendations.

### 5.2 Implications for Research

Future research should focus on long‐term clinical outcomes, particularly implant survival rates and the incidence of peri‐implant disease, to better assess the practical and palpable clinical benefits of dCAIS and sCAIS. Randomized controlled trials (RCTs) with larger sample sizes and standardized methodologies are necessary to reduce heterogeneity and strengthen the evidence base. Further studies should also investigate the impact of different drilling protocols, navigation systems, and measurement methods on transfer accuracy. Research exploring the integration of augmented reality with dynamic navigation systems could offer valuable insights into improving clinical usability and accuracy. Finally, non‐radiological methods for assessing transfer accuracy should be prioritized to reduce patient exposure to additional radiation. Cost‐effectiveness, availability as well as patient‐reported outcomes should also be considered in these trials.

## Author Contributions

F.S.R., M.K. designed the study. F.S.R. and A.K. performed the review. F.S.R. and C.R. performed the search and data extraction. F.S.R. performed the meta‐analysis. F.S.R. and S.R. performed the risk of bias assessment. A.K. functioned as third reviewer in all stages. F.S.R., M.K., A.K., C.R. and S.R. contributed to writing the manuscript. All authors read and approved the final manuscript.

## Disclosure

Registration: The review was prospectively registered on PROSPERO: International Prospective Register of Systematic Reviews database (CRD42024545267 registered on 22 May 2024 and CRD42024545259 registered on 09 June 2024).

## Conflicts of Interest

The first author (F.S.R.) is employed by a company (Straumann GmbH, Germany) that offers computer‐assisted navigation systems. However, F.S.R. has stated that there are no conflicts of interest and there has been no financial support related to this work. The other four co‐authors are not affiliated with this company and have also confirmed that there are no conflicts of interest associated with this publication and there has been no financial support for this work. Moritz Kebschull serves as an Associate Editor of the Journal of Periodontal Research and is a co‐author of this article. In accordance with Wiley’s standard policies for submissions by Editors, he was excluded from the editorial decision‐making related to this article and remained blinded throughout the peer‐review process.

## Supporting information


**Figure S1:** All other forest plots.

**Figure S2:** All funnel plots.

**Figure S3:** All other forest plots.

**Figure S4:** All other forest plots.

**Figure S5:** All other funnel plots.

**Figure S6:** RCT only funnel plots.

**Appendix S1:** Search term.

**Appendix S2:** PRISMA checklist.

**Appendix S3:** Exclusion reasons.

## Data Availability

The authors have nothing to report.
