# Supplementary material for: Accuracy of Freehand, Static, and Dynamic Computer‐Assisted Implant Placement: A Systematic Review and Meta‐Analysis
Source: J Periodontal Res. 2025 Nov 27;61(2):111–37. doi: 10.1111/jre.70047 (PMC12982944; doi:10.1111/jre.70047)
Supplement: Supplementary file 1 — Figure S1: All other forest plots. [file JRE-61-111-s003.pdf]

A

Study	Experimental Mean	Experimental SD	Experimental Total	Control Mean	Control SD	Control Total	Weight	Mean Difference IV, Random, 95% CI
Zhou, M. et al. (2021)	0.40	0.4100	40	1.15	0.3400	40	9.1%	-0.75 [-0.92; -0.58]
Wang, M. et al. (2022)	0.70	0.2900	40	1.17	0.2200	40	9.3%	-0.47 [-0.58; -0.36]
Fang, Q. et al. (2024	0.53	0.1500	60	0.56	0.0600	60	9.4%	-0.03 [-0.07; 0.01]
Shusterman, A. et al. (2024)	0.33	0.1600	45	0.36	0.1200	45	9.4%	-0.03 [-0.09; 0.03]
Mediavalla Guzman, A. et al. (2019)	0.85	0.4800	20	0.78	0.4300	20	8.6%	0.07 [-0.21; 0.35]
Franchina, A. et al. (2020)	0.89	0.3700	30	0.79	0.3500	30	9.0%	0.10 [-0.08; 0.28]
Otaghsara, S. et al. (2023)	0.88	0.3200	20	0.77	0.3200	20	9.0%	0.11 [-0.09; 0.31]
Stunkel, R. et al. (2022)	0.84	0.2600	30	0.65	0.2400	30	9.2%	0.19 [0.06; 0.32]
Werny, J. G., et al. (2025)	1.14	0.7000	48	0.67	0.3200	48	8.9%	0.47 [0.25; 0.69]
Struwe, M. et al. (2023)	1.38	0.8100	180	0.69	0.3000	90	9.2%	0.69 [0.56; 0.82]
Wang, W. et al. (2023)	1.95	0.8000	75	1.05	0.5700	75	8.9%	0.90 [0.68; 1.12]
**Total (95% CI)**			**588**			**498**	**100.0%**	**0.11 [-0.17; 0.39]**

Heterogeneity: $Tau^2 = 0.2132$; $Chi^2 = 343.55$, df = 10 ($P < 0.01$); $I^2 = 97\%$

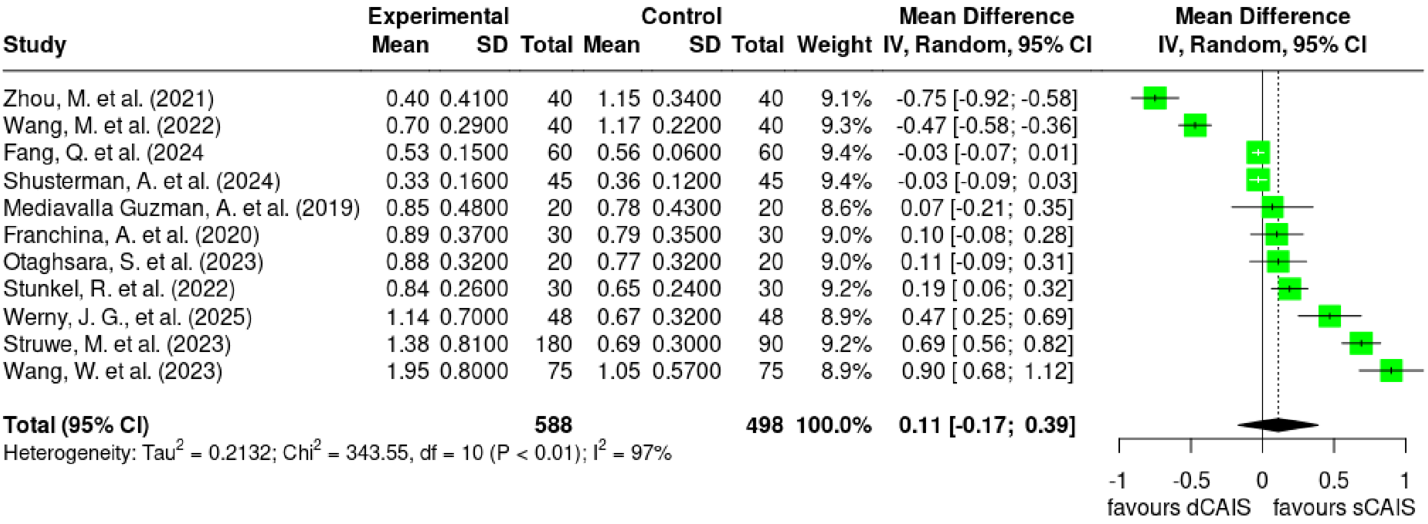

B

Study	Experimental Mean	Experimental SD	Experimental Total	Control Mean	Control SD	Control Total	Weight	Mean Difference IV, Random, 95% CI
Zhou, M. et al. (2021)	0.34	0.3300	40	1.37	0.3800	40	9.3%	-1.03 [-1.19; -0.87]
Wang, M. et al. (2022)	0.88	0.3500	40	1.55	0.3200	40	9.4%	-0.67 [-0.82; -0.52]
Shusterman, A. et al. (2024)	0.40	0.1600	45	0.49	0.1200	45	9.6%	-0.09 [-0.15; -0.03]
Fang, Q. et al. (2024	0.51	0.1000	60	0.56	0.0800	60	9.6%	-0.05 [-0.08; -0.02]
Mediavalla Guzman, A. et al. (2019)	1.18	0.6000	20	1.20	0.6000	20	8.2%	-0.02 [-0.39; 0.35]
Otaghsara, S. et al. (2023)	1.28	0.5500	20	1.26	0.3900	20	8.7%	0.02 [-0.28; 0.32]
Stunkel, R. et al. (2022)	1.01	0.2600	30	0.94	0.5400	30	9.1%	0.07 [-0.14; 0.28]
Franchina, A. et al. (2020)	1.31	0.6800	30	1.17	0.4800	30	8.7%	0.14 [-0.16; 0.44]
Werny, J. G., et al. (2025)	1.23	0.5800	48	1.00	0.3900	48	9.2%	0.23 [0.03; 0.43]
Struwe, M. et al. (2023)	1.41	0.8100	180	1.08	0.4800	90	9.4%	0.33 [0.18; 0.48]
Wang, W. et al. (2023)	2.28	0.9200	75	1.65	0.6600	75	8.9%	0.63 [0.37; 0.89]
**Total (95% CI)**			**588**			**498**	**100.0%**	**-0.05 [-0.32; 0.23]**

Heterogeneity: $Tau^2 = 0.2032$; $Chi^2 = 278.36$, df = 10 ($P < 0.01$); $I^2 = 96\%$

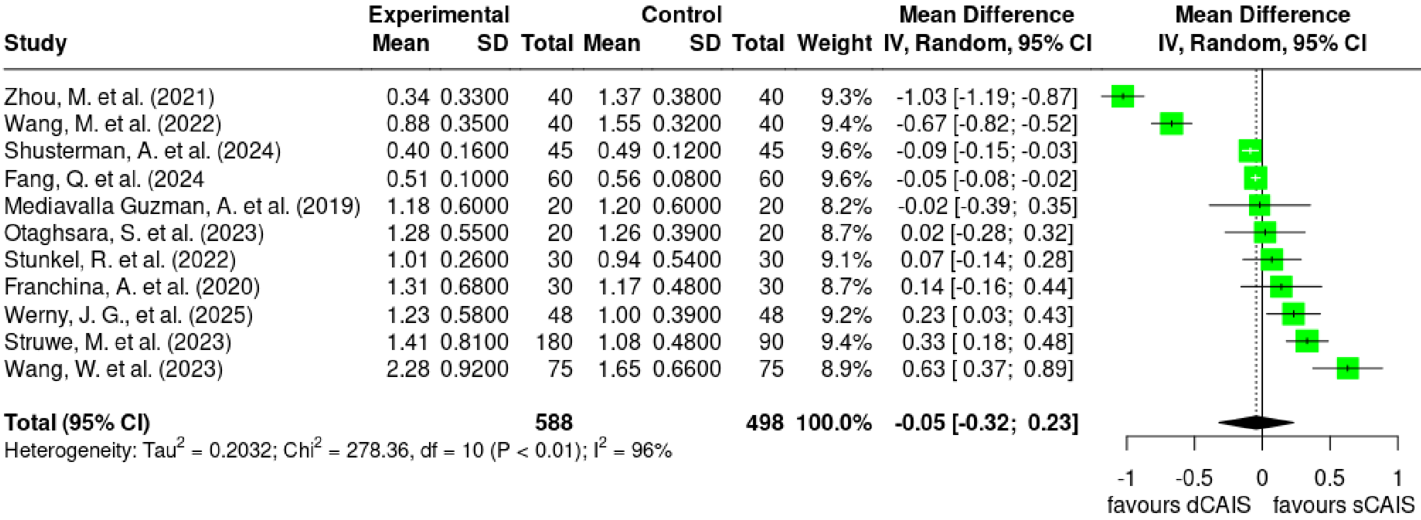

Forest plots demonstrate the analysed transfer accuracy for PICO 1 comparison of dCAIS vs. sCAIS across the deviation parameters: (A) global coronal deviation in millimeters, and (B) global apical deviation in millimeters.

A

Study	Experimental Mean	Experimental SD	Experimental Total	Control Mean	Control SD	Control Total	Weight	Mean Difference IV, Random, 95% CI
Franchina, A. et al. (2020)	3.23	1.0000	30	7.41	3.8700	30	8.1%	-4.18 [-5.61; -2.75]
Werny, J. G., et al. (2025)	2.66	1.7700	48	6.67	3.7000	48	8.7%	-4.01 [-5.17; -2.85]
Abduo, J. and D. Lau (2023)	1.95	1.2500	28	5.83	3.5000	28	8.2%	-3.88 [-5.26; -2.50]
Abduo, J. and D. Lau (2021)	1.42	0.9900	28	5.17	3.0100	28	8.7%	-3.75 [-4.92; -2.58]
Chen, Z., et al. (2022)	1.97	0.9900	100	5.58	2.7400	100	9.9%	-3.61 [-4.18; -3.04]
Guentsch, A. et al. (2021)	1.54	0.5700	60	4.86	2.1000	20	9.3%	-3.32 [-4.25; -2.39]
Shusterman, A. et al. (2024)	1.20	0.3900	45	4.43	1.7200	45	10.0%	-3.23 [-3.75; -2.71]
Abduo, J. et al. (2025)	1.71	1.0800	30	4.62	3.8200	30	8.1%	-2.91 [-4.33; -1.49]
Abduo, J. and D. Lau (2020)	2.52	1.1100	20	4.78	2.0900	20	9.0%	-2.26 [-3.30; -1.22]
Pruthi, G. et al. (2025)	2.51	0.4100	30	3.79	0.6700	30	10.3%	-1.28 [-1.56; -1.00]
Pitman, J., et al. (2024)	3.05	0.9700	24	2.60	1.4100	24	9.7%	0.45 [-0.23; 1.13]
**Total (95% CI)**			**443**			**403**	**100.0%**	**-2.85 [-3.71; -1.98]**

Heterogeneity: $Tau^2 = 1.8688$; $Chi^2 = 171.76$, df = 10 ($P < 0.01$); $I^2 = 94\%$

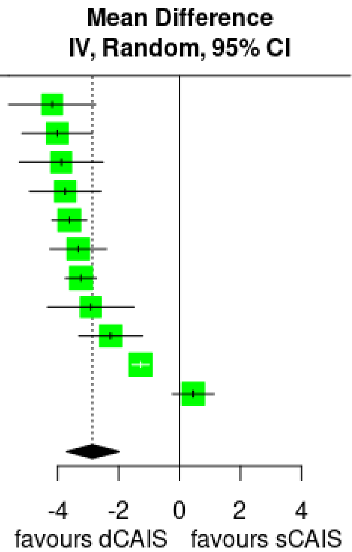

B

Study	Experimental Mean	Experimental SD	Experimental Total	Control Mean	Control SD	Control Total	Weight	Mean Difference IV, Random, 95% CI
Abduo, J. and D. Lau (2021)	0.43	0.2400	28	1.86	0.7000	28	8.6%	-1.43 [-1.70; -1.16]
Franchina, A. et al. (2020)	0.79	0.3500	30	1.65	0.6100	30	8.7%	-0.86 [-1.11; -0.61]
Shusterman, A. et al. (2024)	0.36	0.1200	45	1.08	0.4100	45	9.5%	-0.72 [-0.84; -0.60]
Werny, J. G., et al. (2025)	0.67	0.3200	48	1.31	0.8800	48	8.6%	-0.64 [-0.90; -0.38]
Abduo, J. and D. Lau (2023)	0.44	0.2300	28	1.05	0.6000	28	8.8%	-0.61 [-0.85; -0.37]
Abduo, J. et al. (2025)	0.28	0.1800	30	0.85	0.5100	30	9.1%	-0.57 [-0.76; -0.38]
Abduo, J. and D. Lau (2020)	0.50	0.2600	20	1.03	0.2400	20	9.3%	-0.53 [-0.69; -0.37]
Guentsch, A. et al. (2021)	0.37	0.1300	60	0.80	0.3500	20	9.3%	-0.43 [-0.59; -0.27]
Chen, Z., et al. (2022)	0.57	0.1600	100	0.96	0.4300	100	9.6%	-0.39 [-0.48; -0.30]
Pruthi, G. et al. (2025)	1.31	0.1900	30	1.62	0.3500	30	9.4%	-0.31 [-0.45; -0.17]
Pitman, J., et al. (2024)	0.81	0.2100	24	0.59	0.5000	24	9.0%	0.22 [0.00; 0.44]
**Total (95% CI)**			**443**			**403**	**100.0%**	**-0.56 [-0.79; -0.33]**

Heterogeneity: $Tau^2 = 0.1399$; $Chi^2 = 122.17$, df = 10 ($P < 0.01$); $I^2 = 92\%$

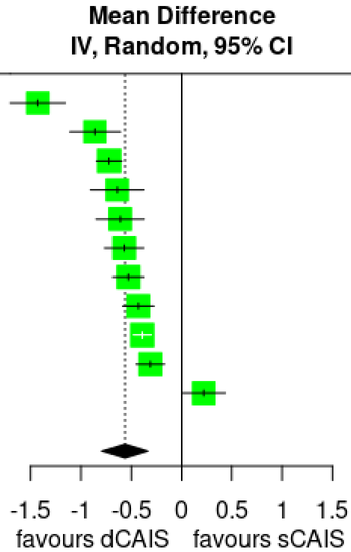

C

Study	Experimental Mean	Experimental SD	Experimental Total	Control Mean	Control SD	Control Total	Weight	Mean Difference IV, Random, 95% CI
Abduo, J. and D. Lau (2021)	0.67	0.4200	28	2.59	1.0300	28	8.6%	-1.92 [-2.33; -1.51]
Franchina, A. et al. (2020)	1.17	0.4800	30	2.33	1.0100	30	8.7%	-1.16 [-1.56; -0.76]
Shusterman, A. et al. (2024)	0.49	0.1200	45	1.53	0.6600	45	9.6%	-1.04 [-1.24; -0.84]
Guentsch, A. et al. (2021)	0.56	0.1700	60	1.53	0.7400	20	9.1%	-0.97 [-1.30; -0.64]
Chen, Z., et al. (2022)	0.93	0.3300	100	1.73	0.7100	100	9.8%	-0.80 [-0.95; -0.65]
Werny, J. G., et al. (2025)	1.00	0.3900	48	1.75	0.9000	48	9.3%	-0.75 [-1.03; -0.47]
Abduo, J. et al. (2025)	0.55	0.3300	30	1.26	0.6400	30	9.4%	-0.71 [-0.97; -0.45]
Abduo, J. and D. Lau (2023)	0.85	0.4200	28	1.48	0.9000	28	8.9%	-0.63 [-1.00; -0.26]
Pruthi, G. et al. (2025)	1.79	0.4700	30	2.40	0.7300	30	9.2%	-0.61 [-0.92; -0.30]
Abduo, J. and D. Lau (2020)	0.73	0.2400	20	0.92	1.1700	20	8.0%	-0.19 [-0.71; 0.33]
Pitman, J., et al. (2024)	1.47	0.3100	24	1.13	0.5400	24	9.4%	0.34 [0.09; 0.59]
**Total (95% CI)**			**443**			**403**	**100.0%**	**-0.76 [-1.09; -0.44]**

Heterogeneity: $Tau^2 = 0.2814$; $Chi^2 = 124.75$, df = 10 ($P < 0.01$); $I^2 = 92\%$

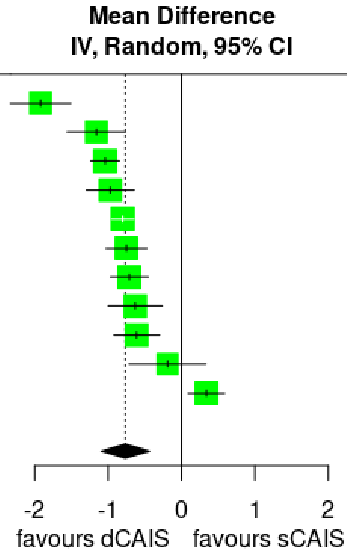

Forest plots demonstrate the analysed transfer accuracy for PICO 1 comparison of sCAIS vs. Freehand across the three deviation parameters: (A) axial deviation in degrees, (B) global coronal deviation in millimeters, and (C) global apical deviation in millimeters.

(A)

Study	Experimental Mean	SD	Total	Control Mean	SD	Total	Weight	Mean Difference IV, Random, 95% CI
Franchina, A. et al. (2020)	2.76	1.3800	30	7.41	3.8700	30	20.2%	-4.65 [-6.12; -3.18]
Shusterman, A. et al. (2024)	0.85	0.3200	45	4.43	1.7200	45	31.0%	-3.58 [-4.09; -3.07]
Werny, J. G., et al. (2025)	3.20	2.1600	48	6.67	3.7000	48	23.1%	-3.47 [-4.68; -2.26]
Mampilly, M. et al. (2023)	7.16	1.7600	30	9.06	2.1800	30	25.6%	-1.90 [-2.90; -0.90]
**Total (95% CI)**			**153**			**153**	**100.0%**	**-3.34 [-4.39; -2.29]**

Heterogeneity: $Tau^2 = 0.8541$; $Chi^2 = 11.84$, df = 3 ($P < 0.01$); $I^2 = 75\%$

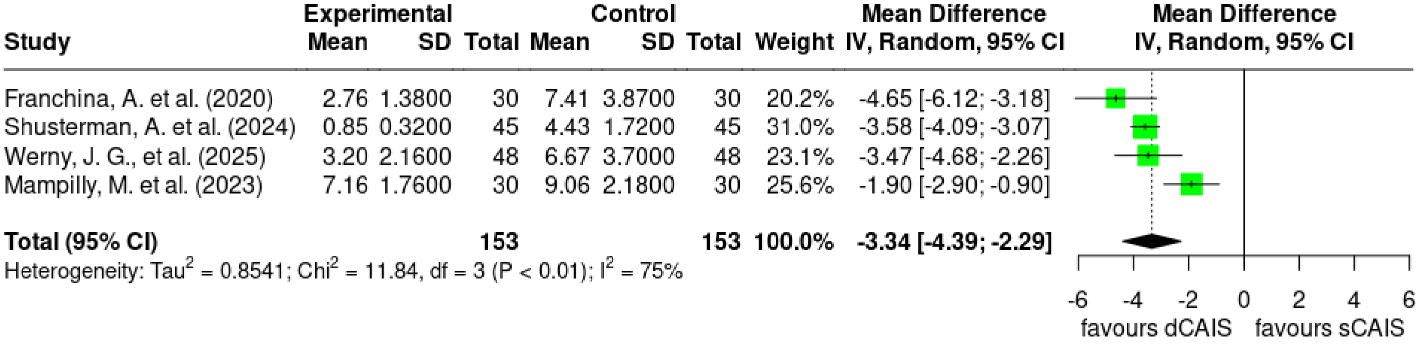

(B)

Study	Experimental Mean	SD	Total	Control Mean	SD	Total	Weight	Mean Difference IV, Random, 95% CI
Mampilly, M. et al. (2023)	5.34	1.4500	30	6.19	3.1400	30	6.0%	-0.85 [-2.09; 0.39]
Franchina, A. et al. (2020)	0.89	0.3700	30	1.65	0.6100	30	30.7%	-0.76 [-1.02; -0.50]
Shusterman, A. et al. (2024)	0.33	0.1600	45	1.08	0.4100	45	35.5%	-0.75 [-0.88; -0.62]
Werny, J. G. et al. (2025)	1.14	0.7000	48	1.31	0.8800	48	27.8%	-0.17 [-0.49; 0.15]
**Total (95% CI)**			**153**			**153**	**100.0%**	**-0.60 [-0.93; -0.27]**

Heterogeneity: $Tau^2 = 0.0755$; $Chi^2 = 11.45$, df = 3 ($P < 0.01$); $I^2 = 74\%$

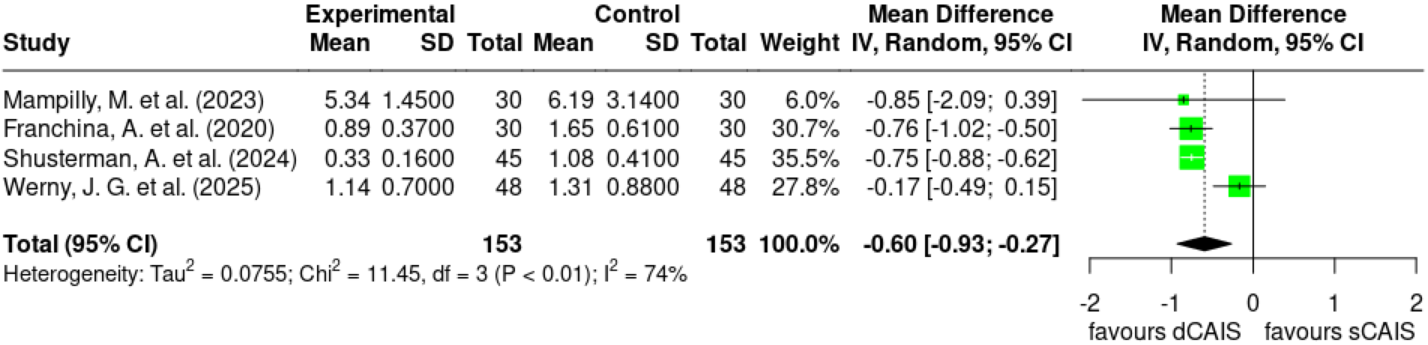

(C)

Study	Experimental Mean	SD	Total	Control Mean	SD	Total	Weight	Mean Difference IV, Random, 95% CI
Shusterman, A. et al. (2024)	0.40	0.1600	45	1.53	0.6600	45	35.1%	-1.13 [-1.33; -0.93]
Mampilly, M., et al. (2023)	5.89	1.0800	30	6.95	2.1200	30	11.1%	-1.06 [-1.91; -0.21]
Franchina, A. et al. (2020)	1.31	0.6800	30	2.33	1.0100	30	23.8%	-1.02 [-1.46; -0.58]
Werny, J. G., et al. (2025)	1.23	0.5800	48	1.75	0.9000	48	30.1%	-0.52 [-0.82; -0.22]
**Total (95% CI)**			**153**			**153**	**100.0%**	**-0.91 [-1.25; -0.58]**

Heterogeneity: $Tau^2 = 0.0719$; $Chi^2 = 11.05$, df = 3 ($P = 0.01$); $I^2 = 73\%$

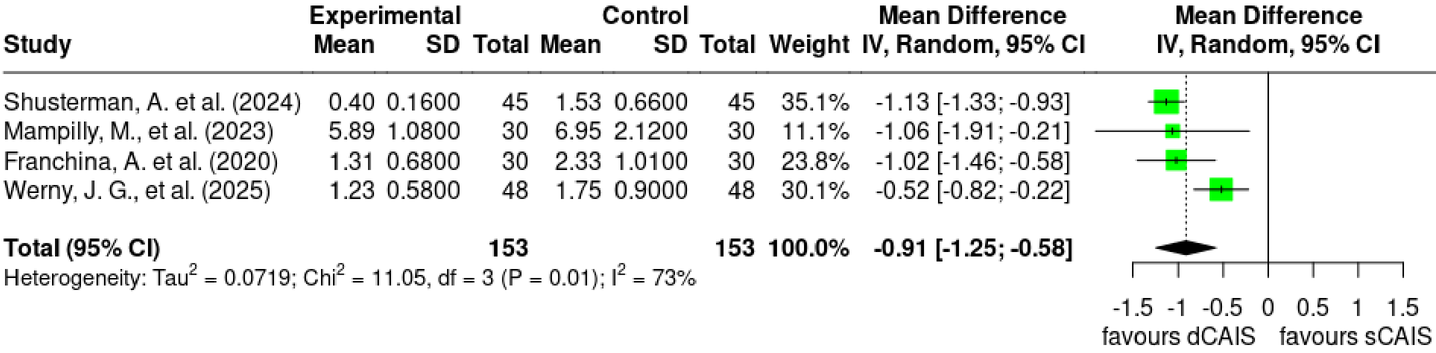

Forest plots demonstrate the analysed transfer accuracy for PICO 1 comparison of dCAIS vs. Freehand across the three deviation parameters: (A) axial deviation in degrees, (B) global coronal deviation in millimeters, and (C) global apical deviation in millimeters.
