# Supplementary material for: Accuracy of Freehand, Static, and Dynamic Computer‐Assisted Implant Placement: A Systematic Review and Meta‐Analysis
Source: J Periodontal Res. 2025 Nov 27;61(2):111–37. doi: 10.1111/jre.70047 (PMC12982944; doi:10.1111/jre.70047)
Supplement: Supplementary file 2 — Figure S2: All funnel plots. [file JRE-61-111-s004.pdf]

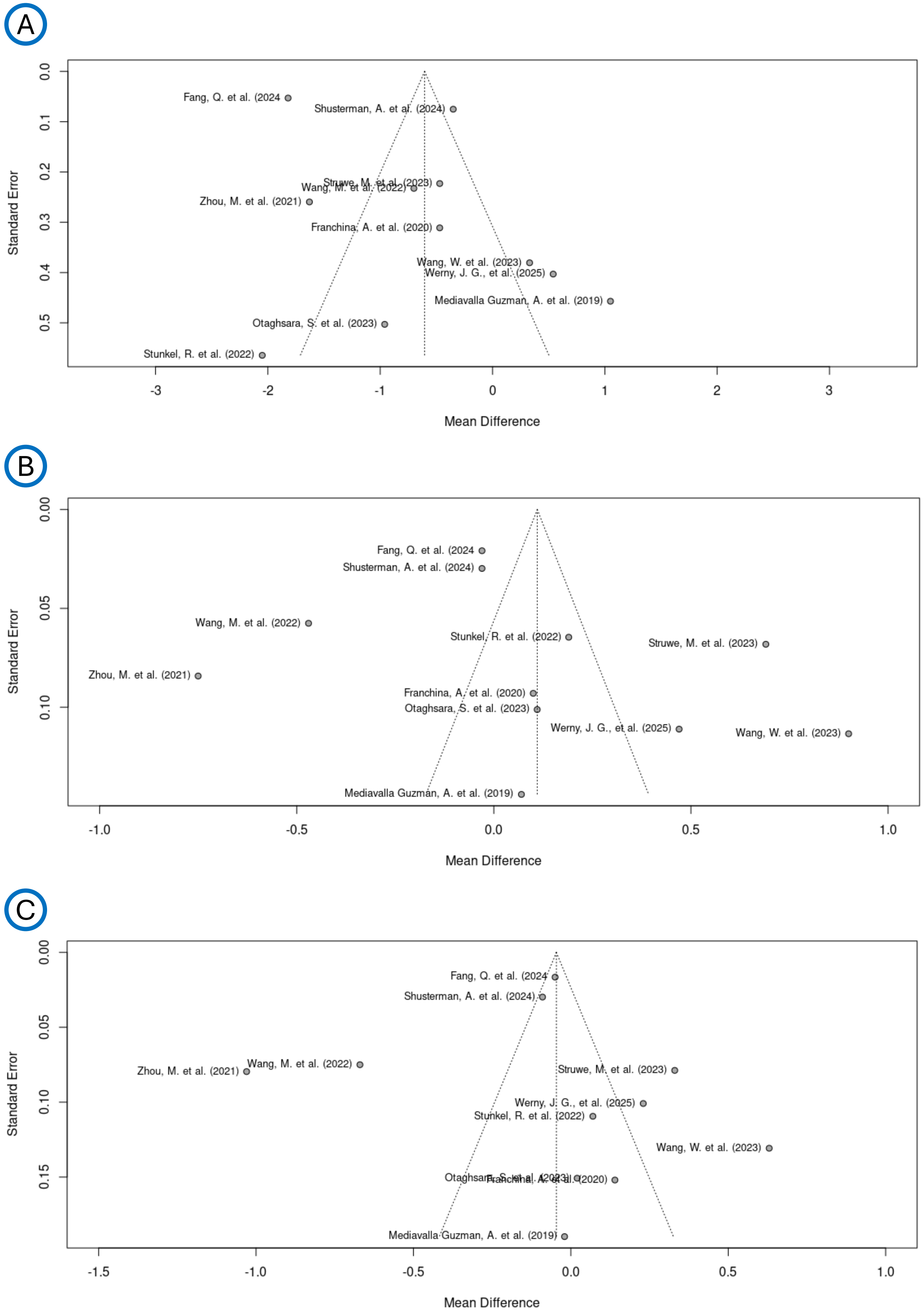

Funnel plots demonstrate the assessment of publication bias for PICO 1, which compares dCAIS versus sCAIS method. The plots investigate the deviation parameters in (A) axial deviation, (B) global coronal deviation, and (C) global apical deviation.

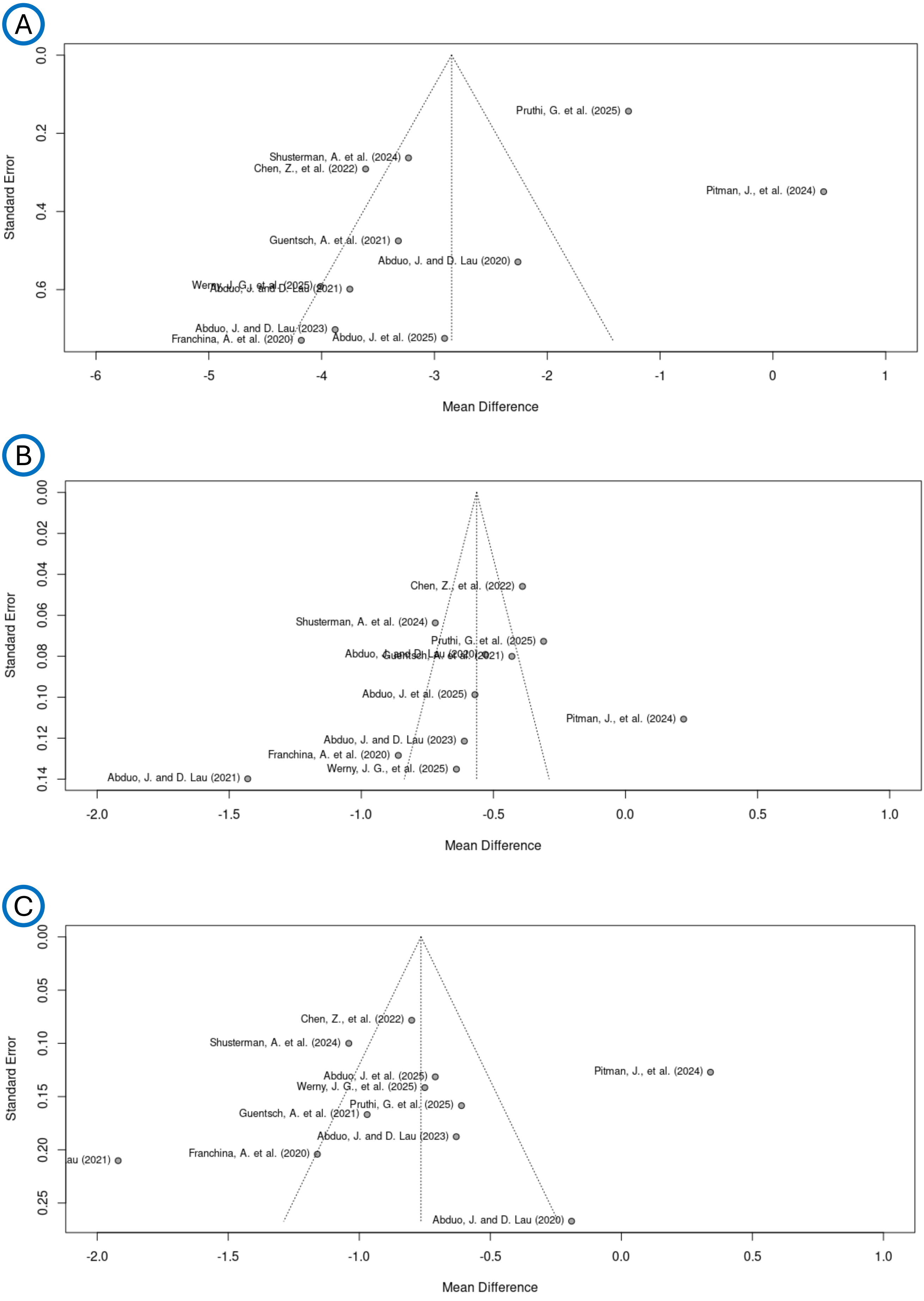

Funnel plots demonstrate the assessment of publication bias for PICO 1, which compares sCAIS versus Freehand method. The plots investigate the deviation parameters in (A) axial deviation, (B) global coronal deviation, and (C) global apical deviation.

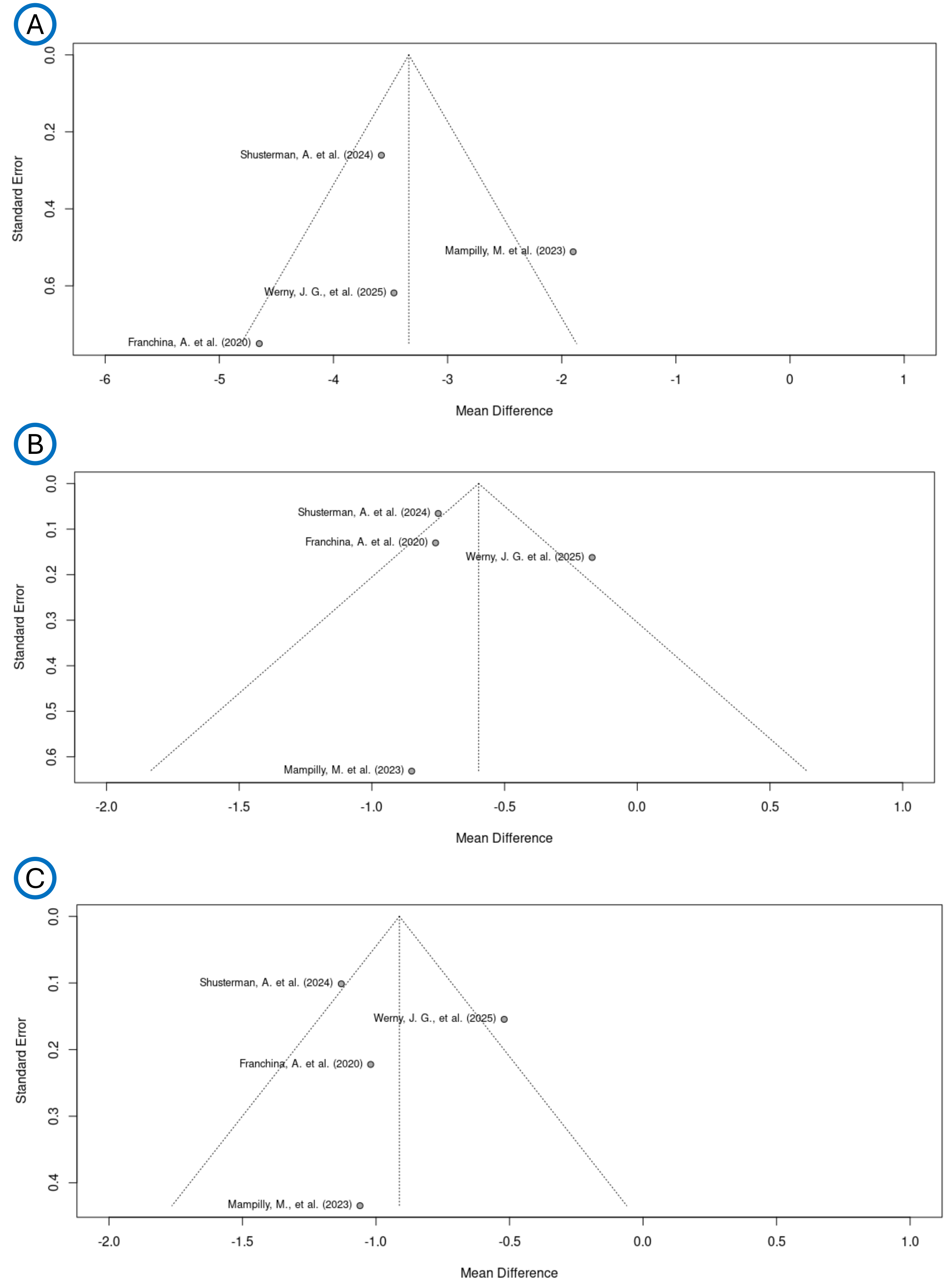

Funnel plots demonstrate the assessment of publication bias for PICO 1, which compares dCAIS versus Freehand method. The plots investigate the deviation parameters in (A) axial deviation, (B) global coronal deviation, and (C) global apical deviation.
