# Supplementary material for: Accuracy of Freehand, Static, and Dynamic Computer‐Assisted Implant Placement: A Systematic Review and Meta‐Analysis
Source: J Periodontal Res. 2025 Nov 27;61(2):111–37. doi: 10.1111/jre.70047 (PMC12982944; doi:10.1111/jre.70047)
Supplement: Supplementary file 3 — Figure S3: All other forest plots. [file JRE-61-111-s006.pdf]

A

Study	Experimental Mean	Experimental SD	Experimental Total	Control Mean	Control SD	Control Total	Weight	Mean Difference IV, Random, 95% CI
Yotpibulwong, T. et al. (2022)	3.18	2.0400	30	7.50	4.0600	30	14.6%	-4.32 [-5.95; -2.69]
Varga, E. et al. (2020)	3.04	1.5100	52	7.03	3.4400	55	38.8%	-3.99 [-4.99; -2.99]
Smitkarn, P. et al. (2019)	3.10	2.3000	30	6.90	4.4000	30	12.2%	-3.80 [-5.58; -2.02]
Younis, H. et al. (2024)	2.52	1.1800	30	5.82	2.7900	30	32.9%	-3.30 [-4.38; -2.22]
Verma, N. et al. (2023)	14.63	8.6900	20	11.89	7.5300	20	1.5%	2.74 [-2.30; 7.78]
**Total (95% CI)**			**162**			**165**	**100.0%**	**-3.69 [-4.31; -3.06]**
**Prediction interval**								**[-4.69; -2.68]**

Heterogeneity: $Tau^2 < 0.0001$; $Chi^2 = 7.69$, df = 4 (P = 0.10); $I^2 = 48\%$

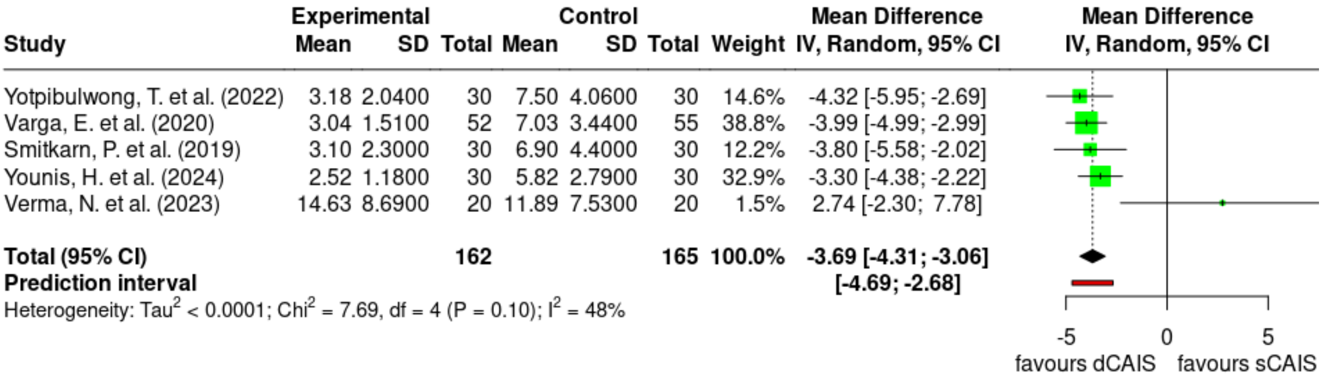

B

Study	Experimental Mean	Experimental SD	Experimental Total	Control Mean	Control SD	Control Total	Weight	Mean Difference IV, Random, 95% CI
Smitkarn, P. et al. (2019)	1.00	0.6000	30	1.50	0.7000	30	19.4%	-0.50 [-0.83; -0.17]
Younis, H. et al. (2024)	0.92	0.3600	30	1.36	0.6200	30	32.0%	-0.44 [-0.70; -0.18]
Varga, E. et al. (2020)	1.40	0.5400	52	1.82	0.9400	55	25.3%	-0.42 [-0.71; -0.13]
Yotpibulwong, T. et al. (2022)	1.06	0.6700	30	1.48	0.6800	30	18.1%	-0.42 [-0.76; -0.08]
Verma, N. et al. (2023)	2.16	0.9200	20	2.00	1.1300	20	5.2%	0.16 [-0.48; 0.80]
**Total (95% CI)**			**162**			**165**	**100.0%**	**-0.41 [-0.56; -0.27]**
**Prediction interval**								**[-0.65; -0.18]**

Heterogeneity: $Tau^2 < 0.0001$; $Chi^2 = 3.41$, df = 4 (P = 0.49); $I^2 = 0\%$

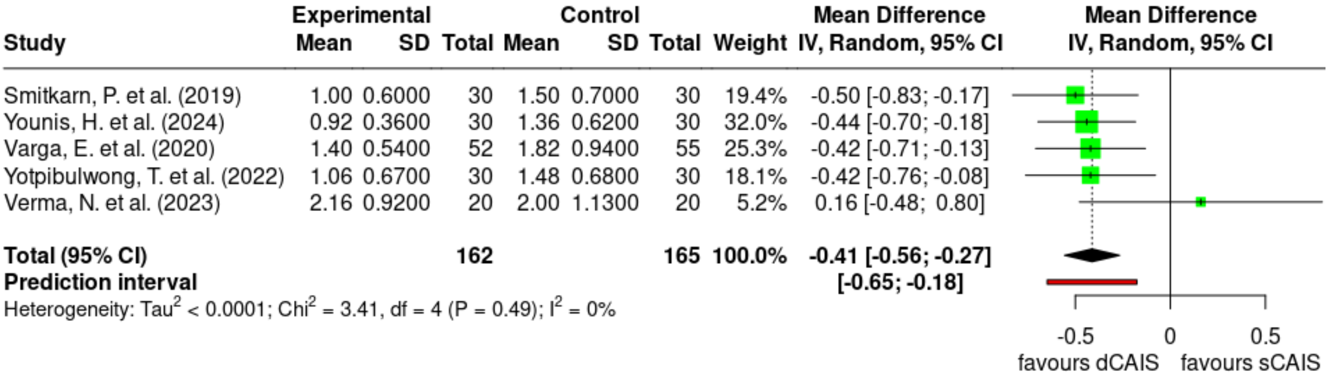

C

Study	Experimental Mean	Experimental SD	Experimental Total	Control Mean	Control SD	Control Total	Weight	Mean Difference IV, Random, 95% CI
Varga, E. et al. (2020)	1.59	0.5900	52	2.43	0.9800	55	31.0%	-0.84 [-1.14; -0.54]
Smitkarn, P. et al. (2019)	1.30	0.6000	30	2.10	1.0000	30	16.5%	-0.80 [-1.22; -0.38]
Yotpibulwong, T. et al. (2022)	1.40	0.7100	30	2.18	0.9500	30	16.0%	-0.78 [-1.20; -0.36]
Younis, H. et al. (2024)	1.06	0.4700	30	1.73	0.6600	30	34.2%	-0.67 [-0.96; -0.38]
Verma, N. et al. (2023)	4.08	1.8800	20	3.52	1.6600	20	2.4%	0.56 [-0.54; 1.66]
**Total (95% CI)**			**162**			**165**	**100.0%**	**-0.73 [-0.90; -0.56]**
**Prediction interval**								**[-1.01; -0.46]**

Heterogeneity: $Tau^2 < 0.0001$; $Chi^2 = 6.12$, df = 4 (P = 0.19); $I^2 = 35\%$

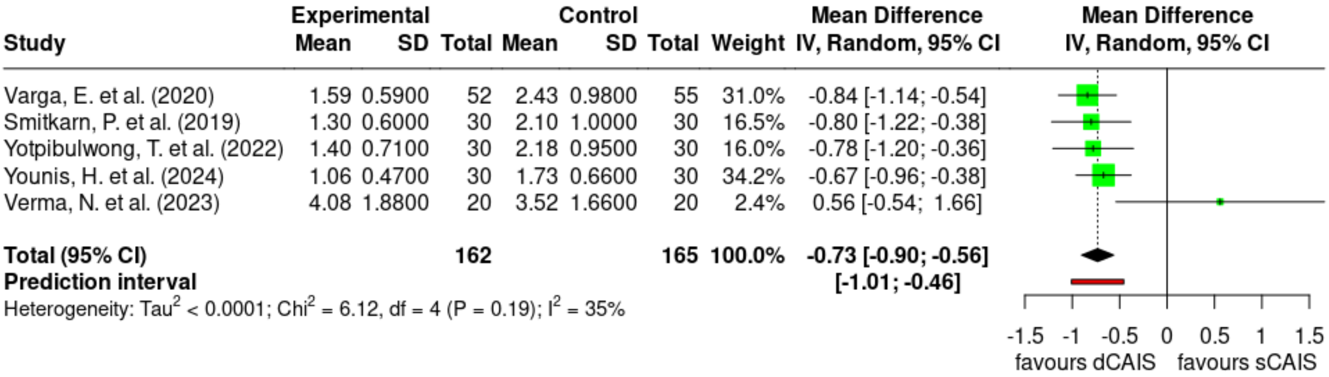

Forest plots demonstrate the analyzed transfer accuracy for PICO 2 with RCT studies only and comparison of sCAIS vs. Freehand across the three deviation parameters: (A) axial deviation in degrees, (B) global coronal deviation in millimeters, and (C) global apical deviation in millimeters.

A

Study	Experimental Mean	Experimental SD	Experimental Total	Control Mean	Control SD	Control Total	Weight	Mean Difference IV, Random, 95% CI
Aydemir, C. A. and V. Arisan (2020)	5.59	0.3900	43	10.40	0.8300	43	28.0%	-4.81 [-5.08; -4.54]
Yotpibulwong, T. et al. (2022)	3.28	1.5700	30	7.50	4.0600	30	20.6%	-4.22 [-5.78; -2.66]
Yang, Y. and N. Geng (2024)	1.03	0.5500	50	3.35	1.1200	46	27.8%	-2.32 [-2.68; -1.96]
Younis, H. et al. (2024)	3.66	1.6400	34	5.82	2.7900	30	23.6%	-2.16 [-3.30; -1.02]
**Total (95% CI)**			**157**			**149**	**100.0%**	**-3.37 [-4.73; -2.01]**
**Prediction interval**								**[-9.73; 2.99]**

Heterogeneity: $Tau^2 = 1.7032$; $Chi^2 = 126.01$, df = 3 ($P < 0.01$); $I^2 = 98\%$

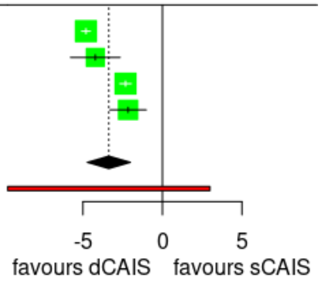

B

Study	Experimental Mean	Experimental SD	Experimental Total	Control Mean	Control SD	Control Total	Weight	Mean Difference IV, Random, 95% CI
Yang, Y. and N. Geng (2024)	0.56	0.0700	50	1.26	0.1300	46	51.0%	-0.70 [-0.74; -0.66]
Aydemir, C. A. and V. Arisan (2020)	1.01	0.0700	43	1.70	0.1300	43	46.8%	-0.69 [-0.73; -0.65]
Yotpibulwong, T. et al. (2022)	1.02	0.4500	30	1.48	0.6800	30	1.1%	-0.46 [-0.75; -0.17]
Younis, H. et al. (2024)	0.99	0.5200	34	1.36	0.6200	30	1.1%	-0.37 [-0.65; -0.09]
**Total (95% CI)**			**157**			**149**	**100.0%**	**-0.69 [-0.72; -0.66]**
**Prediction interval**								**[-0.76; -0.62]**

Heterogeneity: $Tau^2 = 0$; $Chi^2 = 7.53$, df = 3 ($P = 0.06$); $I^2 = 60\%$

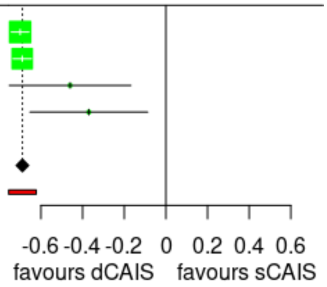

C

Study	Experimental Mean	Experimental SD	Experimental Total	Control Mean	Control SD	Control Total	Weight	Mean Difference IV, Random, 95% CI
Yotpibulwong, T. et al. (2022)	1.28	0.5000	30	2.18	0.9500	30	8.1%	-0.90 [-1.28; -0.52]
Yang, Y. and N. Geng (2024)	0.49	0.2600	50	1.33	0.4200	46	31.8%	-0.84 [-0.98; -0.70]
Aydemir, C. A. and V. Arisan (2020)	1.83	0.1200	43	2.51	0.2100	43	48.1%	-0.68 [-0.75; -0.61]
Younis, H. et al. (2024)	1.14	0.5600	34	1.73	0.6600	30	12.0%	-0.59 [-0.89; -0.29]
**Total (95% CI)**			**157**			**149**	**100.0%**	**-0.74 [-0.86; -0.62]**
**Prediction interval**								**[-1.16; -0.31]**

Heterogeneity: $Tau^2 = 0.0061$; $Chi^2 = 5.46$, df = 3 ($P = 0.14$); $I^2 = 45\%$

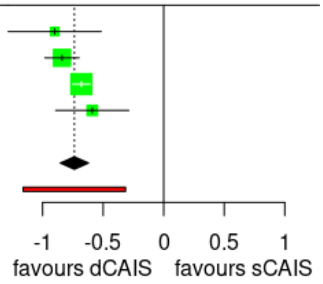

Forest plots demonstrate the analyzed transfer accuracy for PICO 2 with RCT studies only and comparison of dCAIS vs. Freehand across the three deviation parameters: (A) axial deviation in degrees, (B) global coronal deviation in millimeters, and (C) global apical deviation in millimeters.
