# Supplementary material for: Accuracy of Freehand, Static, and Dynamic Computer‐Assisted Implant Placement: A Systematic Review and Meta‐Analysis
Source: J Periodontal Res. 2025 Nov 27;61(2):111–37. doi: 10.1111/jre.70047 (PMC12982944; doi:10.1111/jre.70047)
Supplement: Supplementary file 4 — Figure S4: All other forest plots. [file JRE-61-111-s001.pdf]

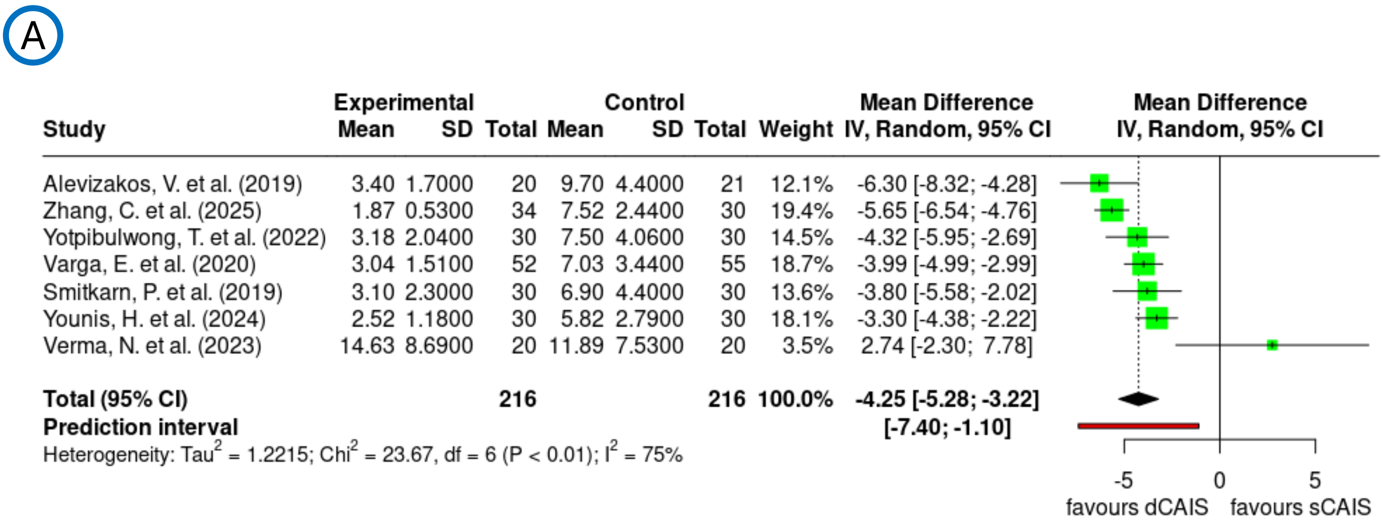

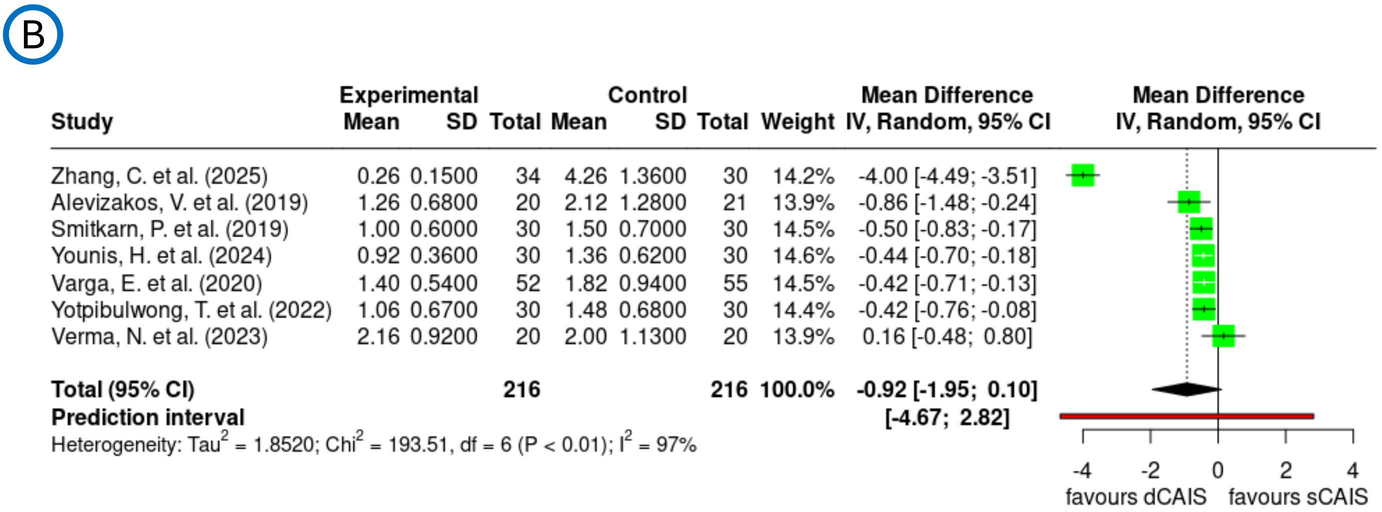

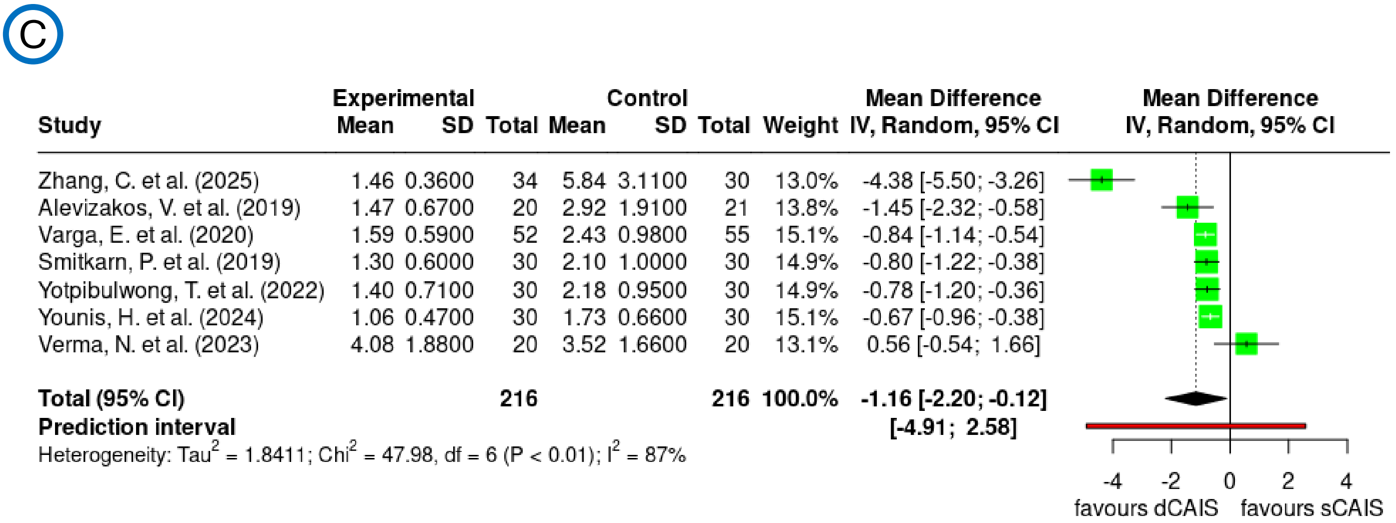

Forest plots demonstrate the analyzed transfer accuracy for PICO 2 comparison of sCAIS vs. Freehand across the three deviation parameters: (A) axial deviation in degrees, (B) global coronal deviation in millimeters, and (C) global apical deviation in millimeters.

A

Study	Experimental Mean	Experimental SD	Experimental Total	Control Mean	Control SD	Control Total	Weight	Mean Difference IV, Random, 95% CI
Zhang, C. et al. (2025)	0.89	0.5800	32	7.52	2.4400	30	16.6%	-6.63 [-7.53; -5.73]
Aydemir, C. A. and V. Arisan (2020)	5.59	0.3900	43	10.40	0.8300	43	17.5%	-4.81 [-5.08; -4.54]
Yotpibulwong, T. et al. (2022)	3.28	1.5700	30	7.50	4.0600	30	15.0%	-4.22 [-5.78; -2.66]
Yang, Y. and N. Geng (2024)	1.03	0.5500	50	3.35	1.1200	46	17.4%	-2.32 [-2.68; -1.96]
Younis, H. et al. (2024)	3.66	1.6400	34	5.82	2.7900	30	16.1%	-2.16 [-3.30; -1.02]
Deng, H. et al. (2025)	1.45	0.3900	38	3.05	1.1700	34	17.4%	-1.60 [-2.01; -1.19]
**Total (95% CI)**			**227**			**213**	**100.0%**	**-3.61 [-5.17; -2.04]**
**Prediction interval**								**[-9.34; 2.13]**

Heterogeneity: $Tau^2 = 3.6292$; $Chi^2 = 264.09$, df = 5 ($P < 0.01$); $I^2 = 98\%$

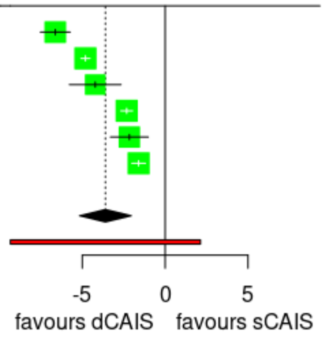

B

Study	Experimental Mean	Experimental SD	Experimental Total	Control Mean	Control SD	Control Total	Weight	Mean Difference IV, Random, 95% CI
Zhang, C. et al. (2025)	0.24	0.1600	32	4.26	1.3600	30	16.3%	-4.02 [-4.51; -3.53]
Deng, H. et al. (2025)	0.43	0.1500	38	1.35	0.5800	34	16.7%	-0.92 [-1.12; -0.72]
Yang, Y. and N. Geng (2024)	0.56	0.0700	50	1.26	0.1300	46	16.8%	-0.70 [-0.74; -0.66]
Aydemir, C. A. and V. Arisan (2020)	1.01	0.0700	43	1.70	0.1300	43	16.8%	-0.69 [-0.73; -0.65]
Yotpibulwong, T. et al. (2022)	1.02	0.4500	30	1.48	0.6800	30	16.6%	-0.46 [-0.75; -0.17]
Younis, H. et al. (2024)	0.99	0.5200	34	1.36	0.6200	30	16.7%	-0.37 [-0.65; -0.09]
**Total (95% CI)**			**227**			**213**	**100.0%**	**-1.18 [-2.28; -0.08]**
**Prediction interval**								**[-5.28; 2.92]**

Heterogeneity: $Tau^2 = 1.8665$; $Chi^2 = 188.97$, df = 5 ($P < 0.01$); $I^2 = 97\%$

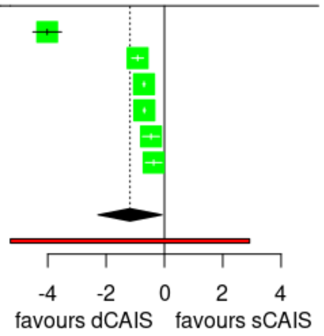

C

Study	Experimental Mean	Experimental SD	Experimental Total	Control Mean	Control SD	Control Total	Weight	Mean Difference IV, Random, 95% CI
Zhang, C. et al. (2025)	0.47	0.3200	32	5.84	3.1100	30	15.4%	-5.37 [-6.49; -4.25]
Yotpibulwong, T. et al. (2022)	1.28	0.5000	30	2.18	0.9500	30	16.8%	-0.90 [-1.28; -0.52]
Yang, Y. and N. Geng (2024)	0.49	0.2600	50	1.33	0.4200	46	17.0%	-0.84 [-0.98; -0.70]
Deng, H. et al. (2025)	0.77	0.2800	38	1.55	1.0300	34	16.8%	-0.78 [-1.14; -0.42]
Aydemir, C. A. and V. Arisan (2020)	1.83	0.1200	43	2.51	0.2100	43	17.0%	-0.68 [-0.75; -0.61]
Younis, H. et al. (2024)	1.14	0.5600	34	1.73	0.6600	30	16.9%	-0.59 [-0.89; -0.29]
**Total (95% CI)**			**227**			**213**	**100.0%**	**-1.47 [-2.88; -0.05]**
**Prediction interval**								**[-6.73; 3.79]**

Heterogeneity: $Tau^2 = 3.0650$; $Chi^2 = 71.94$, df = 5 ($P < 0.01$); $I^2 = 93\%$

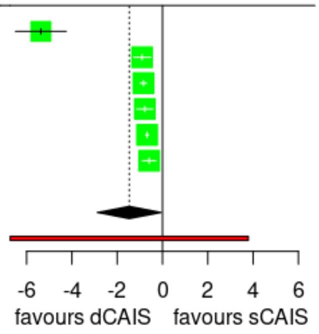

Forest plots demonstrate the analyzed transfer accuracy for PICO 2 comparison of dCAIS vs. Freehand across the three deviation parameters: (A) axial deviation in degrees, (B) global coronal deviation in millimeters, and (C) global apical deviation in millimeters.
