# Supplementary material for: Accuracy of Freehand, Static, and Dynamic Computer‐Assisted Implant Placement: A Systematic Review and Meta‐Analysis
Source: J Periodontal Res. 2025 Nov 27;61(2):111–37. doi: 10.1111/jre.70047 (PMC12982944; doi:10.1111/jre.70047)
Supplement: Supplementary file 7 — Appendix S1: Search term. [file JRE-61-111-s007.pdf]

SM1 *Corresponding search terms used for the electronic literature search.*

MEDLINE (PubMed) - dCAIS vs sCAIS

((((((((((((dental implantation [MeSH Terms]) OR dental implant [MeSH Terms]) AND dental navigation) OR computer aided dental implant) OR three dimensional dental planning) OR 3D dental planning) OR computer assisted dental implant) OR guided dental implant placement) OR dental surgical template) OR dental guided surgery) OR dental surgical guide) OR guided dental implant placement) AND ((((((dynamic) OR augmented reality) OR glasses) OR goggles) OR virtual reality))

MEDLINE (PubMed) - dCAIS vs sCAIS vs freehand

((((((((((((((((((dental implantation [MeSH Terms]) OR dental implant [MeSH Terms]) OR dental navigation) OR computer-aided dental implant) OR three-dimensional dental planning) OR 3D dental planning) OR computer-assisted dental implant) OR guided dental implant placement) OR dental surgical template) OR dental guided surgery) OR dental surgical guide) OR guided dental implant placement) OR dynamic) OR augmented reality) OR glasses) OR goggles) OR virtual reality)) AND (free-hand* OR freehand*)

Google Scholar

"dental implant placement" OR "computer assisted" OR dynamic OR navigation OR guided OR static) AND (free-hand OR "free hand" OR freehand)

Cochrane Library

#1 (dental implant placement):ti,ab,kw

#2 (computer assisted):ti,ab,kw OR (navigation*):ti,ab,kw OR (guided*):ti,ab,kw OR (static*):ti,ab,kw

#3 (free-hand):ti,ab,kw OR (freehand):ti,ab,kw

#4 #1 OR #2 AND #3
