# Supplementary material for: Accuracy of Freehand, Static, and Dynamic Computer‐Assisted Implant Placement: A Systematic Review and Meta‐Analysis
Source: J Periodontal Res. 2025 Nov 27;61(2):111–37. doi: 10.1111/jre.70047 (PMC12982944; doi:10.1111/jre.70047)
Supplement: Supplementary file 9 — Appendix S3: Exclusion reasons. [file JRE-61-111-s005.pdf]

List of excluded studies after full-text assessment

No.	Author / Title	Reason for exclusion
1	Amorfini, L., et al. (2017). "Immediately Loaded Implants in Rehabilitation of the Maxilla: a Two-Year Randomized Clinical Trial of Guided Surgery versus Standard Procedure." Clinical Implant Dentistry and Related Research 19(2): 280-295.	**Does not meet inclusion criteria: Deviating methodology** no camparison of sCAIS with dCAIS and or freehand
2	Amorfini, L., et al. (2023). "Implant rehabilitation of the esthetic area: A five-year retrospective study comparing conventional and fully guided surgery." Clin Implant Dent Relat Res 25(3): 438-446.	**Does not meet inclusion criteria: Relevant outcome not reported** deviation of transfer accuracy not reported
3	Arisan, V., et al. (2013). "Implant positioning errors in freehand and computer-aided placement methods: a single-blind clinical comparative study." Int J Oral Maxillofac Implants 28(1): 190-204.	**Does not meet inclusion criteria: Relevant outcome not reported** deviation of transfer accuracy not reported
4	Aydemir, C. A. and V. Arısan (2020). "Accuracy of dental implant placement via dynamic navigation or the freehand method: a split-mouth randomized controlled clinical trial." Clinical Oral Implants Research 31(3): 255-263.	**Does not meet inclusion criteria: Duplicate**
5	Ayman, D. M., et al. (2022). "Evaluation of Accuracy in Computer Guided Versus Free Hand Immediate Implant Placement in Fresh Extraction Sockets: a Randomized Controlled Clinical Trial." Indian Journal of Public Health Research & Development 13(1).	**Does not meet inclusion criteria: Sample size too small**
6	Baldi, D., et al. (2020). "Digital vs. Freehand Anterior Single-Tooth Implant Restoration." Biomed Res Int 2020: 4012127.	**Does not meet inclusion criteria: Relevant outcome not reported** deviation of transfer accuracy not reported
7	Bernard, L., et al. (2019). "A randomized controlled clinical trial comparing guided with nonguided implant placement: A 3-year follow-up of implant-centered outcomes." J Prosthet Dent 121(6): 904-910.	**Does not meet inclusion criteria: Relevant outcome not reported** deviation of transfer accuracy not reported
8	Bilge, S., et al. (2025). "Comparison of the Accuracy of Pilot Drill-Guided and Fully Guided Implant Surgery With Dynamic Navigation: In Vitro Model Study." J Oral Implantol 51(1): 105-110.	**Does not meet inclusion criteria no full text available**
9	Block, M. S. (2016). "Accuracy Using Static or Dynamic Navigation." J Oral Maxillofac Surg 74(1): 2-3.	**Does not meet inclusion criteria: Study design not met** no comparative study
10	Block, M. S. (2016). "Static and Dynamic Navigation for Dental Implant Placement." J Oral Maxillofac Surg 74(2): 231-233.	**Does not meet inclusion criteria: Study design not met** this study is a report
11	Block, M. S. and R. W. Emery (2016). "Static or Dynamic Navigation for Implant Placement-Choosing the Method of Guidance." J Oral Maxillofac Surg 74(2): 269-277.	**Does not meet inclusion criteria: Study design not met** this study is a report
12	Block, M. S., et al. (2017). "Implant Placement Is More Accurate Using Dynamic Navigation." J Oral Maxillofac Surg 75(7): 1377-1386.	**Does not meet inclusion criteria: Deviating methodology** FH group implants inserted with dynamic navigation but implants were insertet freehand (mix between dCAIS and freehand= unclear method))
13	Brignardello-Petersen, R. (2017). "Small randomized clinical trial shows lack of difference in outcomes when comparing surgically guided and conventional protocol implant placement." Journal of the American Dental Association (1939) 148(6): e75.	**Study design requirements not met This study is a comment** to: SMALL RANDOMIZED CLINICAL TRIAL SHOWS LACK OF DIFFERENCE IN OUTCOMES WHEN COMPARING SURGICALLY GUIDED AND CONVENTIONAL PROTOCOL IMPLANT PLACEMENT Amorfini L, Migliorati M, Drago S, Silvestrini-Biavati A. Immediately loaded implants in rehabilitation of the maxilla: a two-year randomized clinical trial of guided surgery versus standard procedure. Clin Implant Dent Relat Res. 2017;119(2):280-295. http://dx. doi.org/10.1111/cid.12459.

14	Brignardello-Petersen, R. (2019). "Similar deviation between planned and placed implants when using static and dynamic computer-assisted systems in single-tooth implants." J Am Dent Assoc 150(11): e171.	**Does not meet inclusion criteria: no original articel, it´s a comment** N, Pimkhaokham A. The accuracy of static vs. dynamic computer-assisted implant surgery in single tooth space: a randomized controlled trial [published online ahead of print May 6, 2019]. Clin Oral Implants Res. https://doi. org/10.1111/clr.13435.
15	Carrico, C., et al. (2024). "Effect of guided implant placement learning experiences on freehand skills: A pilot study." Clin Exp Dent Res 10(2): e878.	**Does not meet inclusion criteria: Relevant outcome not reported** deviation of transfer accuracy of entry point not in 3D (global) reported
16	Chandran K R, S., et al. (2023). "Accuracy of freehand versus guided immediate implant placement: A randomized controlled trial." Journal of Dentistry 136: 104620.	**Does not meet inclusion criteria: Relevant outcome not reported** deviation of transfer accuracy of entry point not in 3D (global) reported
17	Chandran, K. R. S., et al. (2023). "Accuracy of freehand versus guided immediate implant placement: a randomized controlled trial." Journal of Dentistry 136: 104620.	**Does not meet inclusion criteria: Duplicate**
18	Chen, C. K., et al. (2018). "Accuracy of Implant Placement with a Navigation System, a Laboratory Guide, and Freehand Drilling." Int J Oral Maxillofac Implants 33(6): 1213-1218.	**Does not meet inclusion criteria: Deviating methodology** no implants were inserted, just CBCT of the drill holes has been analysed
19	Chen, Z., et al. (2018). "Accuracy of flapless immediate implant placement in anterior maxilla using computer-assisted versus freehand surgery: A cadaver study." Clin Oral Implants Res 29(12): 1186-1194.	**Does not meet inclusion criteria: Sample size too small**
20	Choi, W., et al. (2017). "Freehand Versus Guided Surgery: Factors Influencing Accuracy of Dental Implant Placement." Implant Dent 26(4): 500-509.	**Does not meet inclusion criteria: Deviating methodology** no comparison between freehand and sCAIS, dCAIS and different measurement methode (transfer accuracy)
21	Deeb, J. G., et al. (2022). "Comparison of Accuracy and Time for Four Implant Placement Techniques Supporting Fixed-Partial Denture." J Oral Implantol 48(6): 562-572.	**Does not meet inclusion criteria: Relevant outcome not reported** The coronal deviation was reported in 2D, no 3D measurements possible due to the navigation system / software
22	Delolme, M. (2022). "Accuracy of implant positioning dynamic: guided surgery VS static guided surgery and freehand surgery."	**Does not meet inclusion criteria no full text available**
23	Duré, M., et al. (2021). "First comparison of a new dynamic navigation system and surgical guides for implantology: an in vitro study." Int J Comput Dent 24(1): 9-17.	**Does not meet inclusion criteria: Duplicate**
24	Duré, M., et al. (2021). "First comparison of a new dynamic navigation system and surgical guides for implantology: an in vitro study." Int J Comput Dent 24(1): 9-17.	**Does not meet inclusion criteria no full text available**
25	Engkawong, S., et al. (2021). "Comparing p atient-reported outcomes and experiences among static, dynamic c omputer-aided, and conventional freehand dental implant placement: A randomized clinical trial." Clinical Implant Dentistry and Related Research 23(5): 660-670.	**Does not meet inclusion criteria: Duplicate**
26	Engkawong, S., et al. (2021). "Comparing patient-reported outcomes and experiences among static, dynamic computer-aided, and conventional freehand dental implant placement: a randomized clinical trial." Clinical Implant Dentistry and Related Research 23(5): 660-670.	**Does not meet inclusion criteria: Relevant outcome not reported** Primary outcome "transfer accuracy" not reported
27	Farley NE, Kennedy K, McGlumphy EA, Clelland NL. Splitmouth comparison of the accuracy of computer- generated and conventional surgical guides. Int J Oral Maxillofac Implants. 2013;28(2):563-572.	**Does not meet inclusion criteria: Sample size too small**
28	Frizzera F, Calazans NNN, Pascoal CH, Martins ME, Mendonça G. Flapless guided implant surgeries compared with conventional surgeries performed by nonexperienced individuals: randomized and controlled Split- mouth clinical trial. Int J Oral Maxillofac Implants. 2021;36(4):755- 761.	**Does not meet inclusion criteria: Sample size too small**
29	Furdin, K. P. S. (2021). "Accuracy between fully guided, half-guided and freehand implant placement on single tooth."	**Does not meet inclusion criteria: Study design not met** not peer reviewed study / no origin article
30	Halabi, D., et al. (2024). "Freehand versus computer-guided implant placement: early implant failures in a case-control study." Quintessence Int 55(7): 548-558.	**Does not meet inclusion criteria: Relevant outcome not reported** transfer accuracy values not reported

31	Hama, D. R. and B. J. Mahmood (2023). "Comparison of accuracy between free-hand and surgical guide implant placement among experienced and non-experienced dental implant practitioners: an in vitro study." Journal of Periodontal & Implant Science 53(5): 388.	**Does not meet inclusion criteria: different measurement methode** (transfer accuracy) and missing tranfer accuracy parameters
32	Hanozin, B., et al. (2022). "Digital vs. conventional workflow for one-abutment one-time immediate restoration in the esthetic zone: a randomized controlled trial." International Journal of Implant Dentistry 8(1): 7.	**Does not meet inclusion criteria: no camparison of sCAIS with dCAIS** and or freehand; sample size too small
33	Heng, S., et al. (2025). "Comparing Medium to Long-Term Esthetic, Clinical, and Patient-Reported Outcomes Between Freehand and Computer-Assisted Dental Implant Placement: A Cross-Sectional Study." J Esthet Restor Dent 37(4): 834-843.	**Does not meet inclusion criteria: Relevant outcome not reported** Primary outcome "transfer accuracy" not reported
34	Heng, S., et al. (2025). "Comparing Medium to Long-Term Esthetic, Clinical, and Patient-Reported Outcomes Between Freehand and Computer-Assisted Dental Implant Placement: A Cross-Sectional Study." Journal of Esthetic and Restorative Dentistry 37(4): 834-843.	**Does not meet inclusion criteria: Duplicate**
35	Huang, L., et al. (2023). "Evaluation of the accuracy of implant placement by using implant positional guide versus freehand: a prospective clinical study." Int J Implant Dent 9(1): 45.	**Does not meet inclusion criteria: Deviating methodology** static Guide was jut use for pilot drill - no fully guided method as typical for sCAIS
36	Hussein, S. K., et al. (2020). "Patient Satisfaction of Computer-Guided Versus Free-Hand Immediate Implant Placement in Esthetic Zone, A Randomized Controlled Trial." Indian Journal of Public Health Research & Development 11(12): 190-196.	**Does not meet inclusion criteria: Relevant outcome not reported** Primary outcome "transfer accuracy" not reported
37	Jaemsuwan, S., et al. (2023). "Comparison of the accuracy of implant position among freehand implant placement, static and dynamic computer-assisted implant surgery in fully edentulous patients: a non-randomized prospective study." Int J Oral Maxillofac Surg 52(2): 264-271.	**Does not meet inclusion criteria: Sample size too small**
38	Jain, S., et al. (2025). "Accuracy and safety of dynamic navigation vs. freehand approach in indirect sinus lift and immediate implant placement: A split mouth clinical study." J Dent 160: 105866.	**Does not meet inclusion criteria: Deviating methodology** distribution of implants and patients numbers per group not reported
39	Jorba-García, A., et al. (2023). "Accuracy of dental implant placement with or without the use of a dynamic navigation assisted system: A randomized clinical trial." Clin Oral Implants Res 34(5): 438-449.	**Does not meet inclusion criteria: Sample size too small**
40	Jorba-Garcia, A., et al. (2023). "Accuracy of dental implant placement with or without the use of a dynamic navigation assisted system: a randomized clinical trial." Clinical Oral Implants Research 34(5): 438-449.	**Does not meet inclusion criteria: Duplicate**
41	Kanduti, D., et al. (2022). "Comparison of free-hand and three guided implant placement techniques: in-vitro study with novice operators." Journal of clinical periodontology 49: 138.	**Does not meet inclusion criteria: Study design not met** Poster Discussion
42	Kivovics, M., et al. (2022). "Accuracy of dental implant placement using augmented reality-based navigation, static computer assisted implant surgery, and the free-hand method: An in vitro study." J Dent 119: 104070.	**Does not meet inclusion criteria: sample size too small** (8 models)
43	Koochaki, M., et al. (2024). "Assessing the Success Rate of Dental Implants Placed Using Guided Systems in Comparison to Free-hand Systems." Journal of Dentomaxillofacial Radiology, Pathology and Surgery 13(1): 31-36.	**Does not meet inclusion criteria no full text available**
44	Kotb, A., et al. (2020). "Esthetic outcome of computer-guided versus free-hand immediate implant placement in fresh extraction sockets in esthetic zone, a randomized clinical trial." Indian journal of public health research and development 11(12): 140-147.	**Does not meet inclusion criteria: Relevant outcome not reported** Primary outcome "transfer accuracy" not reported
45	KR, S. C., et al. (2023). "Accuracy of freehand versus guided immediate implant placement: A randomized controlled trial." Journal of Dentistry 136: 104620.	**Does not meet inclusion criteria: Duplicate**
46	Li, J., et al. (2022). "Open-sleeve templates for computer-assisted implant surgery at healed or extraction sockets: An in vitro comparison to closed-sleeve guided system and free-hand approach." Clin Oral Implants Res 33(7): 757-767.	**Does not meet inclusion criteria: same dataset / Duplicate**: Chen, Z., et al. (2022). "Does guided level (fully or partially) influence implant placement accuracy at post-extraction sockets and healed sites? An in vitro study." Clin Oral Investig 26(8): 5449-5458.
47	Liu, L., et al. (2023). "A mixed reality-based navigation method for dental implant navigation method: A pilot study." Comput Biol Med 154: 106568.	**Does not meet inclusion criteria: Deviating methodology** experimental setup with non-commercial componentes and deviating methodology
48	Lops, D., et al. (2024). "Guided versus freehand single implant placement: A 3-year parallel randomized clinical trial." J Dent 149: 105317.	**Does not meet inclusion criteria: Relevant outcome not reported** Primary outcome "transfer accuracy" not reported

49	Lorwicheanrung, J., et al. "The accuracy of implant placement using a combination of static and dynamic computer-assisted implant surgery in fully edentulous arches: A prospective controlled clinical study." Clinical Oral Implants Research n/a(n/a).	**Does not meet inclusion criteria: Duplicate**
50	Lorwicheanrung, J., et al. (2024). "The accuracy of implant placement using a combination of static and dynamic computer-assisted implant surgery in fully edentulous arches: A prospective controlled clinical study." Clin Oral Implants Res 35(8): 841-853.	**Does not meet inclusion criteria: Sample size too small**
51	Lysenko, A. V., et al. (2023). "Comparison of Dental Implant Placement Accuracy Using a Static Surgical Guide, a Virtual Guide and a Manual Placement Method - An In-Vitro Study." Ann Maxillofac Surg 13(2): 158-162.	**Does not meet inclusion criteria: Relevant outcome not reported** Transfer accuracy not reported at coronal and apical not reported
52	Ma, H., et al. (2021). "Computer-assisted versus traditional freehand technique for mandibular reconstruction with free vascularized fibular flap: A matched-pair study." J Plast Reconstr Aesthet Surg 74(11): 3031-3039.	**Does not meet inclusion criteria: Deviating methodology** no implant placement
53	Magrin GL, Rafael SNF, Passoni BB, et al. Clinical and tomographic comparison of dental implants placed by guided virtual surgery versus conventional technique: a split- mouth randomized clinical trial. J Clin Periodontol. 2020;47(1):120- 128.	**Does not meet inclusion criteria: Sample size too small**
54	Mahmoud, N. R., et al. (2024). "Computer guided versus freehand dental implant surgery: Randomized controlled clinical trial." Saudi Dent J 36(11): 1472-1476.	**Does not meet inclusion criteria: Relevant outcome not reported**
55	Mischkowski, R. A., et al. (2006). "Comparison of static and dynamic computer-assisted guidance methods in implantology." Int J Comput Dent 9(1): 23-35.	**Does not meet inclusion criteria: no full-text available**
56	Mollersten, L. (1989). "Comparison between guided and freehand preparation." The Journal of Prosthetic Dentistry 62(2): 130-139.	**Does not meet inclusion criteria: Deviating methodology** no implant placement
57	Moser, M., et al. (2019). "Accuracy of Patient-Specific Template-Guided Versus Freehand Cervical Pedicle Screw Placement from C2 to C7: A Randomized Cadaveric Study." World Neurosurg 126: e803-e813.	**Does not meet inclusion criteria: Deviating methodology** no standrad dental implant placement
58	Neuschitzer, M., et al. (2025). "Comparative accuracy of dCAIS and freehand techniques for immediate implant placement in the maxillary aesthetic zone: An in vitro study." J Dent 153: 105472.	**Does not meet inclusion criteria: Sample size too small**
59	Nickenig, H.-J., et al. (2010). "Evaluation of the difference in accuracy between implant placement by virtual planning data and surgical guide templates versus the conventional free-hand method–a combined in vivo–in vitro technique using cone-beam CT (Part II)." Journal of Cranio-Maxillofacial Surgery 38(7): 488-493.	**Does not meet inclusion criteria: Sample size too small**
60	Nirula, P., et al. (2023). "Feedback on dental implants with dynamic navigation versus freehand." Bioinformation 19(3): 290-294.	**Does not meet inclusion criteria: Relevant outcome not reported** Transfer Accuracy not reported
61	Ozden Yuce, M., et al. (2020). "Clinical benefits and effectiveness of static computer-aided implant surgery compared with conventional freehand method for single-tooth implant placement." Journal of Stomatology, Oral and Maxillofacial Surgery 121(5): 534-538.	**Does not meet inclusion criteria: Sample size too small**
62	Parekar, D., et al. (2024). "Comparative Evaluation of Accuracy of Adjacent Parallel Implant Placements Between Dynamic Navigation and Static Guide: A Prospective Study." Cureus 16(3): e57331.	**Does not meet inclusion criteria: Relevant outcome not reported** Transfer Accuracy not reported
63	Pomares-Puig, C., et al. (2022). "Dynamic and static computer-guided surgery using the double-factor technique for completely edentulous patients: A dental technique." J Prosthet Dent 128(5): 852-857.	**Does not meet inclusion criteria: Deviating methodology** usin double-factor; no comparison between sCAIS, dCAIS, Freehand
64	Pozzi, A., et al. (2014). "Computer-guided versus free-hand placement of immediately loaded dental implants: 1-year post-loading results of a multicentre randomised controlled trial." Eur J Oral Implantol 7(3): 229-242.	**Does not meet inclusion criteria: Relevant outcome not reported** Transfer Accuracy not reported
65	Sancho-Puchades, M., et al. (2019). "A Randomized Controlled Clinical Trial Comparing Conventional And Computer-Assisted Implant Planning and Placement in Partially Edentulous Patients. Part 2: patient Related Outcome Measures." International journal of periodontics & restorative dentistry 39(4): e99.	**Does not meet inclusion criteria: Relevant outcome not reported** Transfer Accuracy not reported
66	Scherer, U., et al. (2015). "Template-guided vs. non-guided drilling in site preparation of dental implants." Clin Oral Investig 19(6): 1339-1346.	**Does not meet inclusion criteria: Deviating methodology** animal cadaver study
67	Schneider, D., et al. (2018). "A Randomized Controlled Clinical Trial Comparing Conventional and Computer-Assisted Implant Planning and Placement in Partially Edentulous Patients. Part 1: clinician-Related Outcome Measures." International journal of periodontics & restorative dentistry 38(Suppl): s49-s57.	**Does not meet inclusion criteria: Relevant outcome not reported** Transfer Accuracy not reported
68	Schneider, D., et al. (2019). "A Randomized Controlled Clinical Trial Comparing Conventional and Computer-Assisted Implant Planning and Placement in Partially Edentulous Patients. Part 4: accuracy of Implant Placement." International journal of periodontics & restorative dentistry 39(4): e111-e122.	**Does not meet inclusion criteria: Deviating methodology** distribution of implants to groups not reported

69	Schneider, D., et al. (2021). "Accuracy of computer-assisted, template-guided implant placement compared with conventional implant placement by hand-An in vitro study." Clin Oral Implants Res 32(9): 1052-1060.	**Does not meet inclusion criteria: Deviating methodology** deviation of transfer accuracy of entry and apex as "lateral" not not in 3D (global) reported
70	Shi, J. Y., et al. (2025). "Comparison of Implant Precision with Robots, Navigation, or Static Guides." Journal of dental research 104(1): 37-44.	**Does not meet inclusion criteria: Sample size too small**
71	Smitkarn, P., et al. (2019). "The accuracy of single-tooth implants placed using fully digital-guided surgery and freehand implant surgery." Journal of clinical periodontology 46(9): 949-957.	**Does not meet inclusion criteria: Duplicate**
72	Su, T., et al. (2024). "Comparing the accuracies of freehand, static computer-assisted and robot-assisted dental implant placements: an in vitro study." Int J Comput Dent 0(0): 0.	**Does not meet inclusion criteria: Deviating methodology** no jaw models were used. Implant placement in bovine femur & polyurethan subjects
73	Sukhtankar, L. (2019). Evaluation of Accuracy of Static Computer-Guided Implant Placement Compared with Partially-Guided or Free-Hand Placement, Marquette University.	**Does not meet inclusion criteria: Study design not met** no rogin article /compartive study - Master thesis - not peer reviewed study
74	Sun, T. M., et al. (2020). "Comparing Accuracy of Implant Installation with a Navigation System (NS), a Laboratory Guide (LG), NS with LG, and Freehand Drilling." Int J Environ Res Public Health 17(6).	**Does not meet inclusion criteria: Relevant outcome not reported** not all of the deviation parameters reported
75	Tallarico, M., et al. (2018). "Computer-guided vs freehand placement of immediately loaded dental implants: 5-year postloading results of a randomised controlled trial." European journal of oral implantology 11(2): 203-213.	**Does not meet inclusion criteria: Relevant outcome not reported** Primary outcome "transfer accuracy" not reported
76	Tan, P. L. B., et al. (2018). "In vitro comparison of guided versus freehand implant placement: use of a new combined TRIOS surface scanning, Implant Studio, CBCT, and stereolithographic virtually planned and guided technique." Int J Comput Dent 21(2): 87-95.	**Does not meet inclusion criteria: Relevant outcome not reported** Mean value and standard deviation of transfer accuracy not reported
77	Teparrukkul, H., et al. (2024). "Training outcomes of novice clinicians in the use of dynamic computer assisted implant surgery: A prospective comparative study." J Dent Sci 19(Suppl 2): S122-s127.	**Does not meet inclusion criteria: Sample size too small;** different method (group, timing, etc.)
78	Vercruyssen, M., et al. (2014). "An RCT comparing patient-centred outcome variables of guided surgery (bone or mucosa supported) with conventional implant placement." Journal of clinical periodontology 41(7): 724-732.	**Does not meet inclusion criteria: Relevant outcome not reported** Transfer Accuracy not reported
79	Vermeulen, J. (2017). "The Accuracy of Implant Placement by Experienced Surgeons: Guided vs Freehand Approach in a Simulated Plastic Model." Int J Oral Maxillofac Implants 32(3): 617-624.	**Does not meet inclusion criteria: Relevant outcome not reported** standard deviation of transfer accuracy values not reported
80	Wang, X., et al. (2022). "Influence of experience on dental implant placement: an in vitro comparison of freehand, static guided and dynamic navigation approaches." Int J Implant Dent 8(1): 42.	**Does not meet inclusion criteria: relevant outcome not reported** The coronal deviation was reported in 2D, no 3D measurements possible due to the navigation system / software
81	Wang, X., et al. (2023). "Performance of novice versus experienced surgeons for dental implant placement with freehand, static guided and dynamic navigation approaches." Sci Rep 13(1): 2598.	**Does not meet inclusion criteria: relevant outcome not reported** The coronal deviation was reported in 2D, no 3D measurements possible due to the navigation system / software
82	Webb, R. (2019). "Navigation vs Freehand EVD placement accuracy and complications." University of Lynchburg DMSc Doctoral Project Assignment Repository 1(4): 70.	**Does not meet inclusion criteria: Study design not met** no rogin article - Literature review
83	Younes, F., et al. (2018). "A randomized controlled study on the accuracy of free-handed, pilot-drill guided and fully guided implant surgery in partially edentulous patients." J Clin Periodontol 45(6): 721-732.	**Does not meet inclusion criteria: Deviating methodology** standard deviation of transfer accuracy values not reported
84	Younes, F., et al. (2019). "A randomized controlled trial on the efficiency of free-handed, pilot-drill guided and fully guided implant surgery in partially edentulous patients." Clin Oral Implants Res 30(2): 131-138.	**Does not meet inclusion criteria: Duplicate= same dataset:** study excluded due to using same data set reported in study: Younes, F., et al. (2018). "A randomized controlled study on the accuracy of free-handed, pilot-drill guided and fully guided implant surgery in partially edentulous patients." J Clin Periodontol 45(6): 721-732.
85	Younis, H., et al. (2024). "Accuracy of dynamic navigation compared to static surgical guides and the freehand approach in implant placement: a prospective clinical study." Head Face Med 20(1): 30.	**Does not meet inclusion criteria: Duplicate**

86	Zhan, Y., et al. (2021). "Evaluation of a dynamic navigation system for training students in dental implant placement." J Dent Educ 85(2): 120-127.	**Does not meet inclusion criteria: Deviating methodology** different method due to severeal training stages and evaluation on different time
87	Zhao, W., et al. (2024). "Accuracy of dental implant surgery with freehand, static computer-aided, dynamic computer-aided, and robotic computer-aided implant systems: An in vitro study." J Prosthet Dent.	**Does not meet inclusion criteria: relevant outcome not reported** standrad deviation at FH protocol not reported
88	Zhong, X., et al. (2024). "Surgical performance of dental students using computer-assisted dynamic navigation and freehand approaches." Eur J Dent Educ 28(2): 504-510.	**Does not meet inclusion criteria: Deviating methodology** different method due to severeal training stages and evaluation on different time
